# Country-scale assessment of urban areas, population, and households exposed to land subsidence using Sentinel-1 InSAR, and GPS time series

**DOI:** 10.1007/s11069-023-06259-5

**Published:** 2023-10-29

**Authors:** Enrique Antonio Fernández-Torres, Enrique Cabral-Cano, Darío Solano-Rojas, Luis Salazar-Tlaczani, Josue Gárcia-Venegas, Bertha Marquez-Azúa, Shannon Graham, Katia Michelle Villarnobo-Gonzalez

**Affiliations:** 1 Posgrado en Ciencias de la Tierra, Universidad Nacional Autónoma de México, Ciudad Universitaria, 04510 Coyoacán, Mexico City, Mexico; 2 Departamento de Geomagnetismo y Exploración, Instituto de Geofísica, Universidad Nacional Autónoma de México, Ciudad Universitaria, 04510 Coyoacán, Mexico City, Mexico; 3 División de Ingeniería en Ciencias de la Tierra, Facultad de Ingeniería, Universidad Nacional Autónoma de México, Ciudad Universitaria, 04510 Coyoacán, Mexico City, Mexico; 4 Centro de Estudios Estratégicos Para el Desarrollo, Universidad de Guadalajara, Ladrón de Guevara, Tomás V. Gómez 121, 44100 Guadalajara, Jalisco Mexico; 5 The College of New Jersey Physics Department, 2000 Pennington Rd, Ewing, NJ 08628 USA; 6 Departamento de Física, Facultad de Ciencias, Universidad Nacional Autónoma de México, Ciudad Universitaria, 04510 Coyoacán, Mexico City, Mexico

**Keywords:** InSAR, Sentinel-1, Urban land subsidence, GPS, Mexico, Nation-wide

## Abstract

The increased need for water resources in urban sprawls and intense droughts has forced more aggressive groundwater extraction resulting in numerous urban areas undergoing land subsidence. In most cases, only some large metropolitan areas have been well-characterized for subsidence. However, there is no existing country-wide assessment of urban areas, population, and households exposed to this process. This research showcases a methodology to systematically evaluate urban localities with land subsidence higher than − 2.8 cm/year throughout Mexico. We used Interferometric Synthetic Aperture Radar (InSAR) tools with a dataset of 4611 scenes from European Space Agency’s Sentinel-1 A/B SAR sensors acquired from descending orbits from September 2018 through October 2019. This dataset was processed at a supercomputer using InSAR Scientific Computing Environment and the Miami InSAR Time Series software in Python software. The quality and calibration of the resulting velocity maps are assessed through a large-scale comparison with observations from 100 continuous GPS sites throughout Mexico. Our results show that an urban area of 3797 km^2^, 6.9 million households, and 17% of the total population in Mexico is exposed to subsidence velocities of faster than − 2.8 cm/year, in more than 853 urban localities within 29 land subsidence regions. We also confirm previous global potential estimations of subsidence occurrence in low relief areas over unconsolidated deposits and where groundwater aquifers are under stress. The presented research demonstrates the capabilities for surveying urban areas exposed to land subsidence at a country-scale level by combining Sentinel-1 velocities with spatial national census data.

**Supplementary Information:**

The online version contains supplementary material available at 10.1007/s11069-023-06259-5.

## Introduction

Since the industrial revolution, the planet has suffered a constant increase in the release of greenhouse gases resulting in an increasing trend in the temperature and changes in the precipitation patterns (IPCC 2021). These changes in climate behavior have had considerable alterations in the hydrological system, causing a rise in the intensity and frequency of floods and droughts. As result of the surface water scarcity, aggressive groundwater extraction rates result in stressed aquifers, and loss of storage capacity that may ultimately land subsidence (Herrera-García et al. 2021). Ground subsidence, a natural or human-induced phenomena caused by underground materials movement producing topographic level decrease (e.g., Galloway et al. 1999), generates substantial economic loss in urban areas due to damage to infrastructure and/or increase flooding probability. In human-induced subsidence, surface lowering mainly due to surface loading and extraction of underground fluids and/or solid materials (Galloway et al. 1999) and its velocity can be ten times higher than the natural surface level decrease (Meckel 2008).

Ground-level decrease due to groundwater extraction occurs as a response to the reduction in pore water stress and the resulting increase in effective pressure, generating compaction of unconsolidated sediments (Carrillo 1948). The physical behavior of land subsidence may be controlled by subsoil composition and stress history, which can have an elastic or inelastic response (Terzaghi 1925). The elastic deformation occurs when the preconsolidation stress in the aquifer system is not exceeded. The preconsolidation stress is the maximum vertical effective stress that the subsoil has suffered due to its own weight or external loading (Casagrande 1936). The preconsolidation stress is influenced by several factors such as environmental conditions, hydrology, secondary compression, chemical alteration, variation in overburden pressure, sedimentation rate, sediment age, and tectonic conditions (e.g., Brumund et al. 1976). Small displacements on the surface characterize the elastic deformation, the deformation is recoverable, and it can have seasonal behavior depending on the water table fluctuations (e.g., Galloway et al. 1999). In contrast, in the inelastic deformation, the aquitard skeleton may suffer significant and permanent reorganization and occurs when the load exceeds the preconsolidation stress. In the inelastic deformation, pore fluids move into the aquifers; as a result, pore volume irreversibly decreases (e.g., Galloway et al. 1999). Inelastic land subsidence can produce very fast land subsidence rates (e.g., Chaussard et al. 2014). In this case, the ground subsidence process has important consequences as aquifer storage capacity severely decreases, eventually restricting the long-term viability of urban areas that depend on aquifers/aquitards for their water supply.

InSAR techniques have been satisfactorily applied to detect land subsidence in many cities worldwide (e.g., Raspini et al. 2022), and the land movement precision measurement mainly depends on the decorrelation of the SAR signal, phase unwrapping errors, and atmospheric delays. To overcome errors related to SAR signal decorrelation, two families of InSAR time series techniques were developed, PS and DS. The PS algorithm is based on the phase-stable point scatterers (PS), which mainly correspond to man-made structures and bare rocks surfaces (e.g., Ferretti et al. 2001). The second family is distributed scatterers (DS); these algorithms include areas with decorrelation using a redundant network of interferograms (e.g., Berardino et al. 2002). Several algorithms are available for reducing unwrapping errors, such as the bridging method, phase closures method, coherence-based network to exclude interferograms with coherent phase unwrapping errors, and iterative spatial bridging (Yunjun et al. 2019; Oliver-Cabrera et al. 2022). In the case of tropospheric delay, we can use the empirical relationship between stratified troposphere delay and topography (Doin et al. 2009) or using global atmospheric models, such as ERA-I (Dee et al. 2011). Moreover, in recent years, modern SAR satellites (e.g., PAZ, ALOS-2/PALSAR-2, Sentinel-1), new InSAR process algorithms, and powerful computation resources are available, allowing to measure land surface deformation at a country-level with high accuracy (Manunta et al. 2019; Morishita 2021).

The ESA’s Sentinel-1 A and B SAR C-band constellation can accurately measure land movement deformation. These sensors have improved capabilities such as an orbital tube of 200 m, revisit time of 6 days (12 for a single sensor), nearly worldwide coverage, free data policy, the TOPS (Terrain Observations by Progressive Scan) image acquisition mode that allows reaching 250 km of swath width, and a spatial resolution of 5 × 20 m^2^ (interferometric wide swath mode; Torres et al. 2012). As a result, Sentinel-1 data have been used for a wide spectrum of applications, including mapping of natural hazards, monitoring of glacier flows, and surveillance of ship routes (Torres et al. 2012). In addition, Sentinel-1 C-band sensors provide data time continuity with previous C-band SAR sensors (i.e., Envisat and ERS missions), making possible to generate InSAR long time series deformation assessment which can include several decades (e.g., Chaussard et al. 2021). Therefore, several studies have used Sentinel-1 data to monitor land movement on a regional scale (e.g., Crosetto et al. 2020). However, a few country-scale studies measure land subsidence occurrence and their spatial patterns, magnitudes, urban areas, population, and households exposed to ground subsidence.

This work is aimed at providing a country-scale assessment of urban areas, population, and households exposed to land subsidence and how they can be grouped into land subsidence regions. Relationships were evaluated between conditional factors (e.g., lithology and groundwater health) and urban areas with land subsidence. Finally, we also provide an extensive quality assessment by comparing InSAR time series and permanent GPS stations.

## Study area

**Fig. 1:**
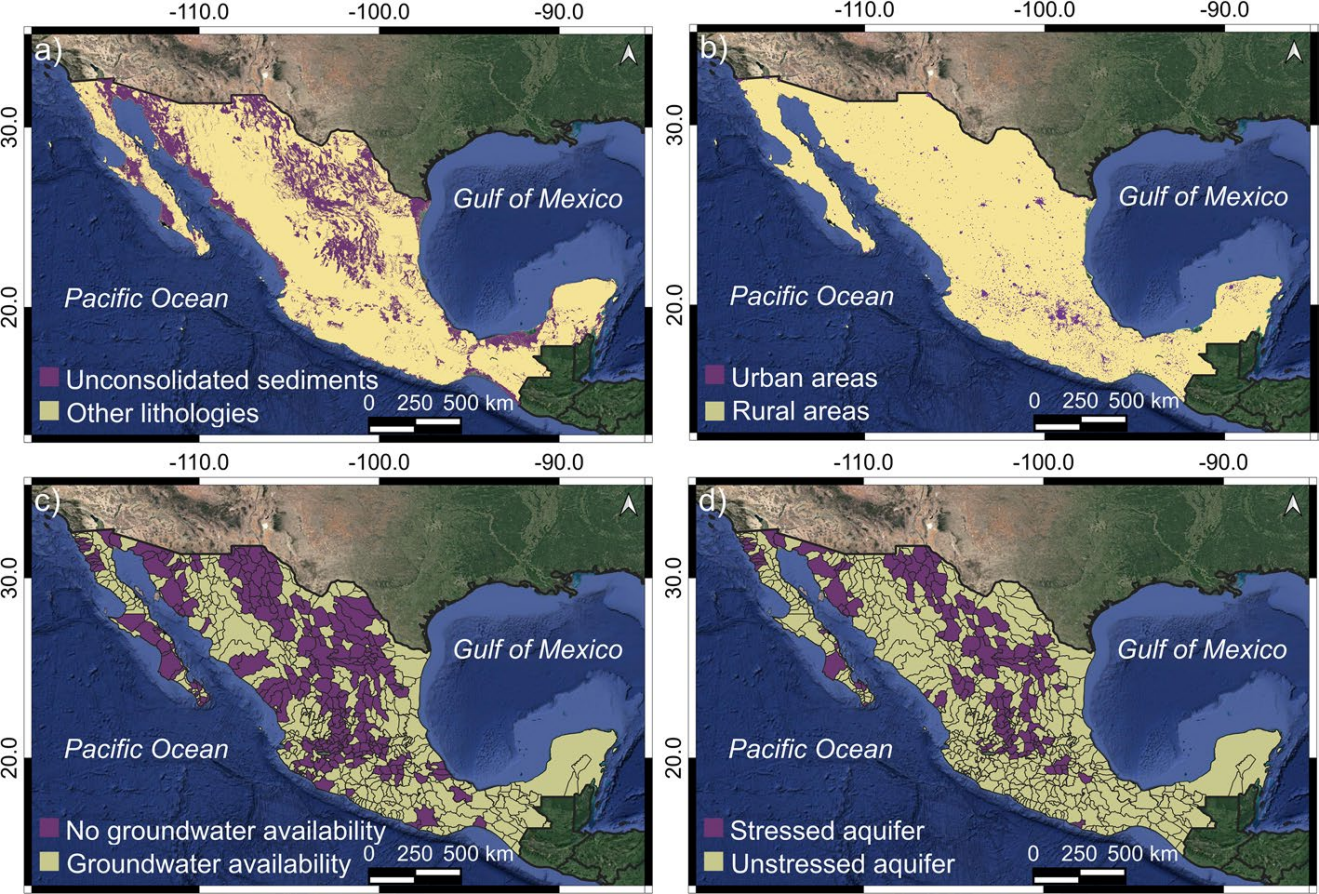
Spatial distribution of factors that define urban areas with land subsidence. **a** Distribution of unconsolidated deposits based on (SGM 1998). **b** Urban AGEBs of Mexico (modified from INEGI 2020). **c** Groundwater availability. **d** Groundwater stress. (Modified from CONAGUA 2020). Google Earth imagery was used as the base map in Figs. 1, 3, 4, 5, and 7, and QGIS 3.22 software to compose the maps (https://www.qgis.org/es/site/)

The main factors that increase the potential of land subsidence due groundwater extraction in Mexico are the spatial distribution of compressible deposits and population density (e.g., Chaussard et al. 2014). As a result, we used the information of the spatial distribution of unconsolidated deposits (Fig. 1a, modified from SGM 1998), the distribution of urban areas (Fig. 1b, modified from INEGI 2020), and groundwater condition (Fig. 1c, d, modified from CONAGUA 2020) to define the potential area to land subsidence.

Mexico lacks cartographic products with thickness, compressibility index, and porosity values of unconsolidated sediments. Consequently, we used a criterion based on surface lithology with the potential to develop land subsidence. This criterion assumes that unconsolidated surface lithology poses high compressibility and porosity values; therefore, there is a high potential for land subsidence velocity (Chaussard et al. 2014). Under this broad category of compressible-unconsolidated deposits, we grouped all alluvial, lacustrine, colluvial, eolian, littoral, and palustrine lithologies from the *Servicio Geológico Mexicano* cartographic database (SGM 1998). Figure 1a shows the spatial distribution of unconsolidated deposits with high compressibility and porosity potential. This figure also shows that unconsolidated-compressible deposits are present in well-defined regions that include a central east–west region, a central north–south and the coastal plains in the Gulf of Mexico, northwestern Mexico, and portions of the Baja California Peninsula (Fig. 1a). In the first two areas, the extensional Cenozoic stress regime favored developing of grabens that promoted the accumulation of unconsolidated-compressible materials (e.g., Henry and Aranda-Gomez 1992; García-Palomo et al. 2000; Nieto-Samaniego et al. 2005).

In Mexico, 79% of the total population live in urban areas (INEGI 2020); moreover, land subsidence is a significant problem in affected urban localities because of the resulting damage to buildings and urban infrastructure. Therefore, in this study, we used the spatial distribution of urban *Áreas Geoestadísticas Básicas* (AGEBs) shapefiles from the 2020 Housing and Population Census (INEGI 2020), as an area of interest. The urban AGEBs are composed of street blocks (usually ~ 1–50) that are grouped in geographically delimited polygons (INEGI 2020; Figs. 1b, 4b) to optimize the nation-wide statistical analysis. Consequently, the higher the density of urban AGEB, the higher the groundwater extraction to cover the population needs. The AGEB spatial distribution shows that most of the urban areas are located in central Mexico along an east–west corridor that coincides with the Mexican Volcanic Belt (Figs. 1b, 4b); subsequently, this area of Mexico has the highest potential to develop land subsidence (Chaussard et al. 2014). However, there are also urban areas that spatially overlap with compressible-unconsolidated deposits, such as the central plateau in northern Mexico and along the northwestern Mexico and Gulf of Mexico coastal areas (Fig. 1a, b).

For the distribution of groundwater, we used *the Comisión Nacional del Agua* groundwater availability and stress map (Fig. 1c, d; CONAGUA 2020). CONAGUA analysis indicates that 205 out of 653 of Mexico’s aquifers have no additional groundwater availability (Fig. 1c). Meanwhile, 105 out of 653 of Mexico’s aquifers have an extraction rate exceeding their natural recharge (CONAGUA 2020, Fig. 1d). Only southeastern Mexico has both groundwater availability and unstressed aquifers (Fig. 1c, d). The region east of the Tehuantepec isthmus currently has a low potential of developing land subsidence associated with groundwater extraction and thus was not considered in our current analysis (Fig. 3). Nevertheless, there are reports of ground subsidence due to hydrocarbon extraction in Villahermosa, Tabasco, in southeastern Mexico (Pérez-Falls and Martínez-Flores 2020) although they are spatially restricted.

Previous research on subsidence by groundwater withdrawal only covered the east–west central Mexico and Cerro Prieto regions [initially described by Carnec and Fabriol (1999), Cabral-Cano et al. (2008), Chaussard et al. (2014)] but at the same time still missing its reconnaissance over broad regions of Mexico with high potential for land subsidence. In consequence, in this research, we evaluated the land subsidence in urban areas located over the intersection of high compressible deposits with stressed aquifers and no additional groundwater available, thus covering large areas of the country that have not been previously analyzed for subsidence.

## Methodology

**Fig. 2:**
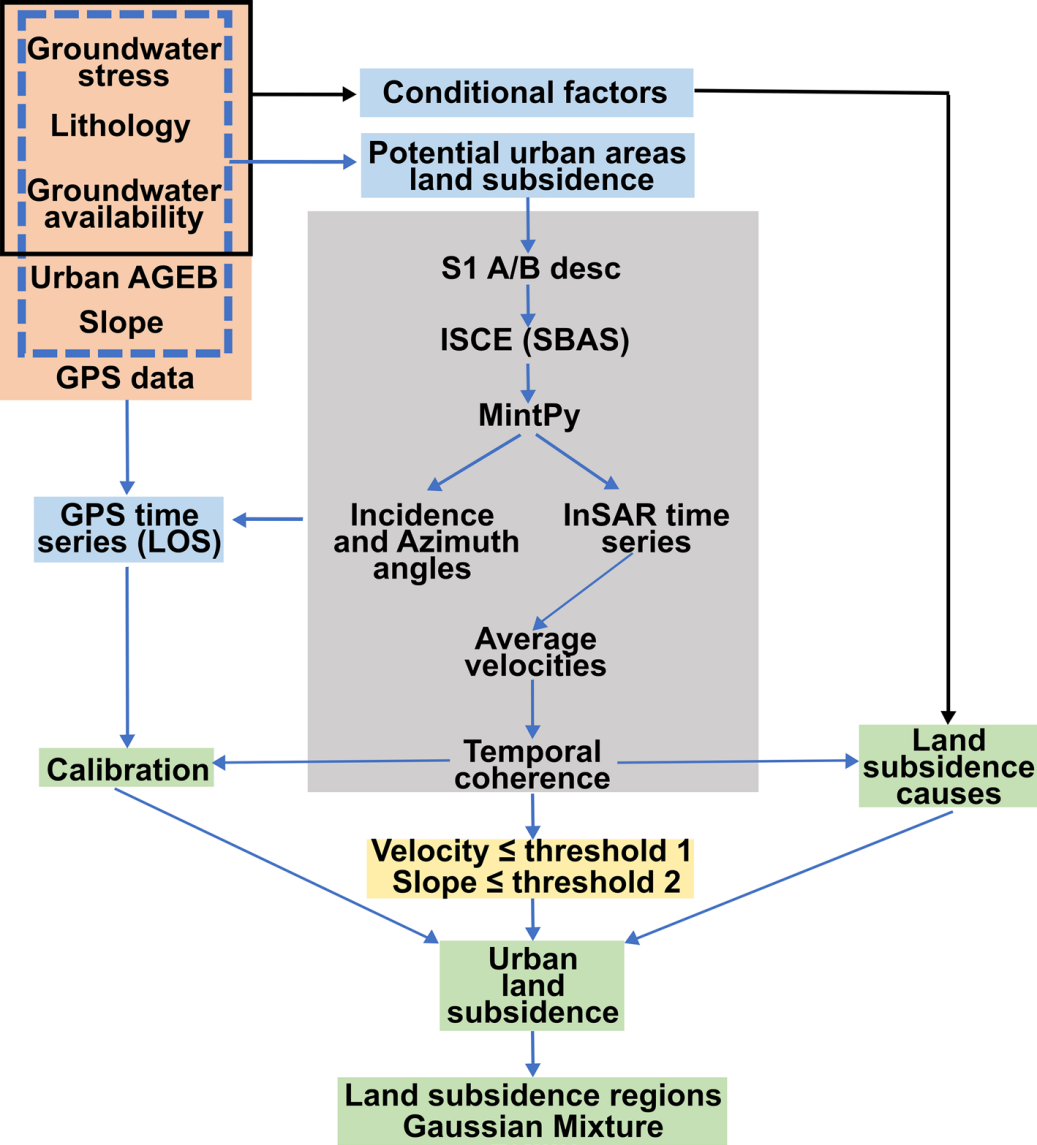
Workflow to detect urban areas with land subsidence. Orange polygon represents input data. Light blue polygons are intermediate products. InSAR-SBAS methodology is inside gray polygon. Yellow polygon has the velocity and slope thresholds 1 and 2, respectively. Light green polygons are final products

To estimate the urban areas exposed to land subsidence in Mexico, we first narrow the area of interest using the spatial distribution of aquifer health (potential urban areas land subsidence; see Sect. 2). We then performed an InSAR analysis to systematically identify and characterize velocity fields and InSAR time series. Subsequently, we applied a temporal coherence mask for quality control, and then thresholds for velocity and slope were utilized. We also overlapped the urban AGEB regions with the InSAR velocity field to find the urban localities with land subsidence which were classified according to their spatial distribution using the Gaussian mixture algorithm (Fig. 2). Then, we analyzed relationships between urban areas with land subsidence and conditional factors (i.e., lithology, aquifer condition, land subsidence causes; Fig. 2). Finally, we compared InSAR and GPS time series velocities to calibrate the accuracy of our results (Fig. 2; calibration).

### SAR dataset and InSAR processing

**Fig. 3:**
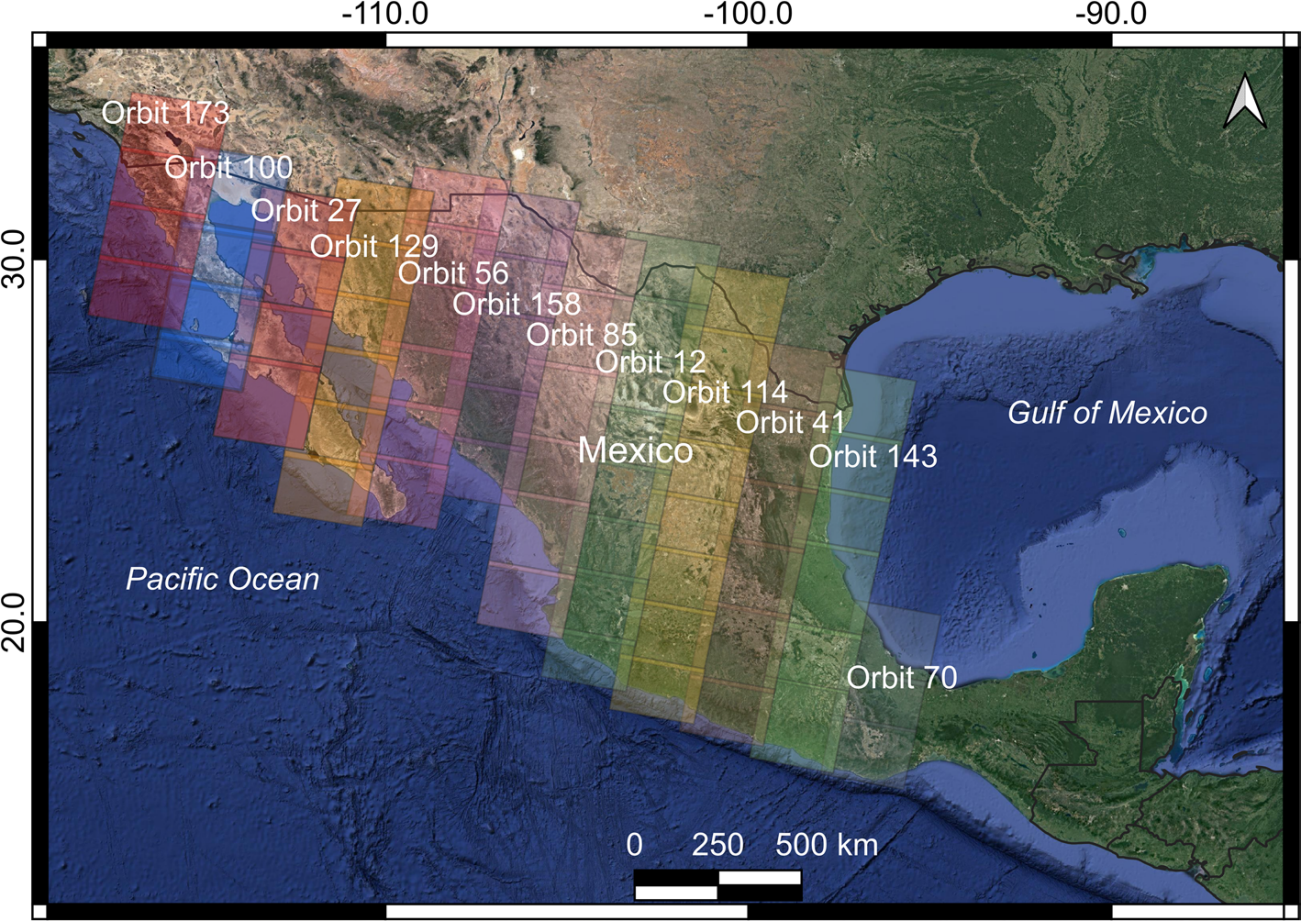
Relative orbits that cover the subsiding areas of interest in descending path from Sentinel-1

**Fig. 4:**
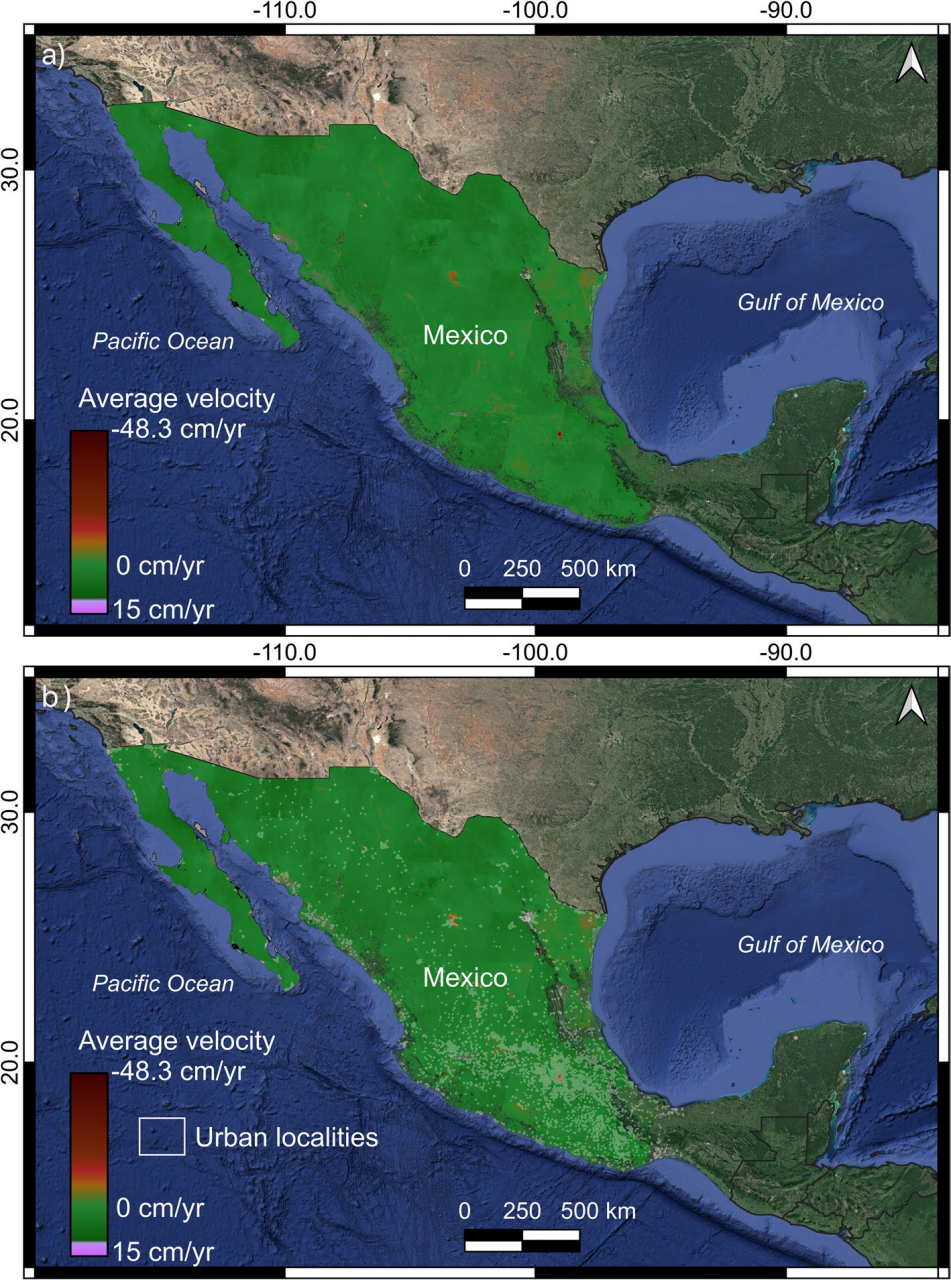
Velocity maps and urban AGBEB of Mexico. **a** Mosaic map of ground subsidence associated with groundwater extraction in Mexico. **b** Average velocity map mosaic with the urban localities’ polygons superimposed

The Synthetic Aperture Radar (SAR) data consists of 4,611 Single Look Complex (SLC) scenes acquired by the Sentinel-1 A/B sensors. The SAR SLC images were obtained from September 1, 2018, to October 30, 2019, along 12 descending orbits (Fig. 3; Table 1) and covering 1.7 million km^2^ (Fig. 4a, b). The SAR scenes were processed with interferometric networks of three connections using the Small Baseline Subset algorithm (Berardino et al. 2002). The InSAR processing was carried out with a multilooking of 20 by 60 looks in azimuth and range orientations, resulting in a size of ~ 300 × 300 m^2^. We used SBAS (DS) algorithm because it used an interferometric network with a small spatial and temporal baseline reaching a higher spatial coverage than PS algorithms and allowing to measure land deformation with millimetric precision over thousands of square kilometers (i.e., regional studies, e.g., Manunta et al. 2019; Morishita 2021). Besides, Sentinel-1 scenes have a small orbital tube (i.e., 200 m) and high revisit time frequency to reduce spatial decorrelation effects.

**Table 1:** Relative orbits, observation periods, and number of dates used in this study

Relative orbit	Date (yyyymmdd)	# SLC	# IFG
	Start–End		
173	20180902–20191027	123	116
100	20180909–20191022	245	198
27	20181115–20191029	105	78
129	20180911–20191024	385	396
56	20180906–20191019	392	396
158	20180901–20191026	403	408
85	20180908–20191021	344	276
12	20180903–20191028	539	405
114	20180910–20191023	455	396
41	20180905–20191030	661	699
143	20180906–20191025	543	480
70	20180901–20191026	416	454
Total		4611	4302

To process the SAR acquisitions, we used the JPL/Caltech ISCE software (Rosen et al. 2012), obtaining 4302 unwrapped interferograms, which were then processed using the MintPy (Yunjun et al. 2019) to compute the average velocity and the deformation time series of each pixel. In this InSAR processing we also applied the network-based enhanced spectral diversity (NESD; Fattahi et al. 2017) and the empirical correlation between troposphere and topography (Doin et al. 2009), for a precise interferogram co-registration and corrected tropospheric errors, respectively. The noisy scenes were estimated using the root-mean-square (RMS) approach from Yunjun et al. (2019) and a temporal coherence mask of 0.7 was implemented to keep only high-quality pixels (Pepe and Lanari 2006).

To compare our results with previous research and considering that in Mexico most of the cGPS the vertical displacement component is higher than the horizontal components (e.g., Blewitt et al. 2018; Cabral-Cano et al. 2018), the resulting InSAR average velocities maps were projected from LOS deformation (*dlos*) into vertical deformations (*dv*) using the incidence angle of the sensors *θ (dv* = *dlos*/cos* θ*), assuming that there is no lateral displacement. We also processed a longer InSAR time series (January 2018–June 2021) in some places with remarkable land subsidence velocities (i.e., Mexico City Metropolitan Area, Jocotepec Jalisco, Torreón Coahuila, and Chaparrosa Zacatecas; Supplementary file 1) to compare with the one-year observation velocities used for the entire country. These more extended InSAR time series were obtained using 1020 Sentinel-1 A/B SLC scenes acquired in descending relative orbits (i.e., 41, 114, and 12). The Sentinel-1 A/B SLC January 2018–June 2021 scenes were processed with the same methodology applied to process the smaller InSAR time series (i.e., September 2018–October 2019).

Due to the size of the data and computer requirements, the InSAR processing of the entire country was divided into forty sub-areas and was performed at a supercomputing facility. We required 19 TB for SAR scenes FTP downloads, 108.1 TB to generate unwrapped interferograms, and 0.1 TB for velocity maps and time series generation (Table 2).

**Table 2:** Supercomputing resources

Parameter	Total storage (TB)	Time (hh:mm)
FTP SLCs	19	57:20
ISCE	108.1	284:01
Mintpy	0.1	07:45
Total	127.2	347:10

### Urban areas exposed to land subsidence and classification of land subsidence regions

To identify urban areas exposed to land subsidence, we overlapped urban AGEBs polygons over the InSAR velocity map (Fig. 4a, b). AGEBs provide the basic unitary information from the INEGI’s national Housing and Population 2020 census, available at street-block scale and with multiple parameters such as population, number of households, total area, states, municipalities, urban localities, and others which can be grouped as urban localities (Fig. 4b). Then, we identify all AGEBs with a maximum subsidence velocity faster than or equal to − 2.8 cm/year.

The velocity threshold is based on the value of 1.5 standard deviations (SD) of velocity pixels of the study area (1.7 million km^2^), following previously defined criteria which pointed out that the SD of velocity is an indicator of the level of noise (e.g., Tomás et al. 2019). Moreover, using a relatively high threshold prevents overestimating the total urban areas exposed to land subsidence. The second threshold, slope values lower than 5 degrees, was used considering that land subsidence occurs where the negative vertical velocity overcomes horizontal rates and over very flat topography (e.g., Tomás et al. 2019).

In the next step, AGEBs of the same states and municipalities were grouped using INEGI’s polygons of urban localities (INEGI 2020). The urban localities were grouped following similar velocity categories used by Chaussard et al. (2014). These categories are low [− 2.8 to − 5 cm/year], intermediate (− 5 to − 10] cm/year, and high with a subsidence rate faster than − 10 cm/year. Next, we measured the areas, population, and households between each category and identified the latitude and longitude of the polygon’s centroid of the AGEB with maximum average subsidence velocity (Supplementary file 2).

The Gaussian mixture model (GMM) was then used to spatially classify urban areas undergoing land subsidence. This probabilistic approach can be implemented as an unsupervised machine learning technique. For the GMM classification, we employed the Scikit-learn machine learning library (Pedregosa et al. 2011). We implemented the GMM over the spatial distribution of land subsidence urban location polygon centroids to group them into clusters (*k*). To determine the optimal number of *k*, we applied the Bayesian information criterion (BIC) over a different number of clusters to find the number of *k* that minimizes the theoretical information criterion. In other words, the number of *k *that better fits our data. We also determined the best value of the covariance type hyperparameter according to the spatial distribution of the data. Our best model parameters (i.e., *k* and covariance type) were then used to fit the GMM to our data (i.e., the latitude and longitude values of land subsidence urban location centroids) and obtained each cluster parameters including weights, means, and covariance matrices of each *K*. Finally, to identify the relationship between urban areas with land subsidence and conditional factors (Fig. 2), we created an intersection table between urban localities with land subsidence and aquifer units (CONAGUA 2020) and lithology (SGM 1998) (Supplementary file 2).

### Calibration

**Fig. 5:**
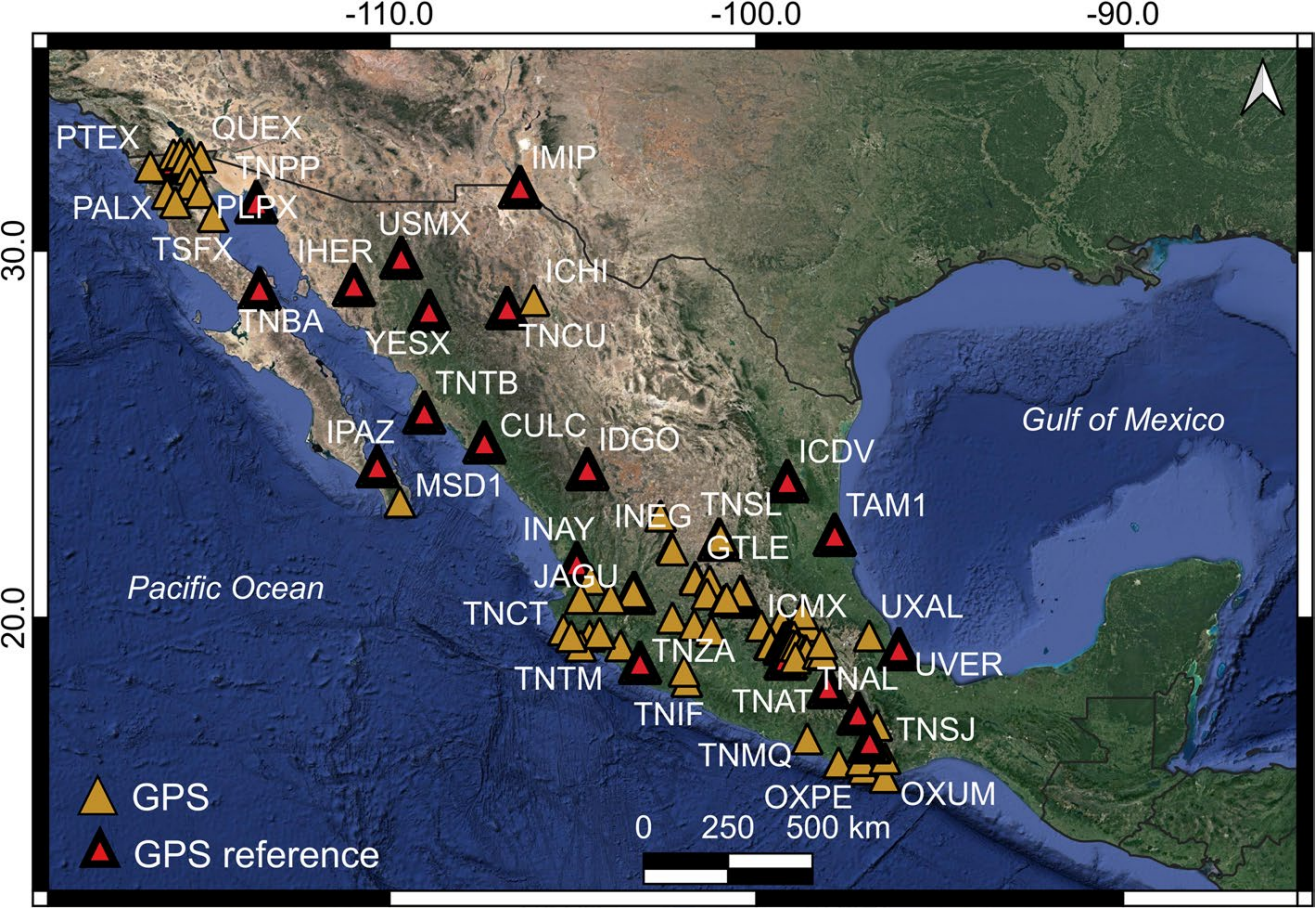
Location map of the 100 continuous GPS stations that include all the TLALOCNet GPS stations (Cabral-Cano et al. 2018) and other time series solution available at the Nevada Geodetic Laboratory (Blewitt et al. 2018 used for calibration and accuracy assessment of the InSAR velocities)

The area of interest was divided into forty sub-areas and where there is at least one continuous GPS station that presents a null vertical velocity that is used as reference points in 34 out of 40 InSAR-processed Sentinel-1 relative orbit zones (Figs. 3, 5). In those six cases where there are no continuous GPS permanent stations available inside the area, we assigned the reference point in a coherence pixel located on a rock outcrop (and presumably) a stable, non-subsiding area.

To investigate the accuracy of the generated InSAR time series, we compared the InSAR time series results with those from 100 continuous GPS stations that include all the TLALOCNet GPS stations (Cabral-Cano et al. 2018) and other time series solutions available at the Nevada Geodetic Laboratory (Blewitt et al. 2018). In this comparison, we considered the following:

1. We used those continuous GPS stations with an average time overlap of 1.03 years with the InSAR time series (see Supplementary file 3).
2. GPS coordinate components time series (i.e., north–south, west–east, and vertical) were projected into line-of-sight direction by considering the InSAR-processed based incidence and azimuthal angles at each GPS’s position (e.g., Catalao et al. 2011).
3. For both continuous GPS and InSAR time series, the average velocity was computed using a linear regression analysis using the time window of InSAR data (September 2018 to October 2019).
4. GPS measurements were assumed as reference.
5. GPS stations are those located in InSAR velocity coherence pixels.

To assess the accuracy, we calculated the difference between cGPS and InSAR time series velocities and measured the correlation between InSAR and GPS velocities (Fig. 11; Table 5).

## Results

### Spatial pattern and time series of land subsidence

The average velocity map mosaic is presented as a vertical component in Fig. 4a. This map displays only pixels with temporal coherence higher than 0.7 providing the time series of 188.8 million coherence pixels and covering an area of about 1.7 million km^2^. Pixels with red colors represent the areas with higher displacement away from the satellite (e.g., subsidence), pixels with green colors velocity represent areas with lower and null displacement away from the satellite, and areas with purple color show areas with movement toward the satellite (e.g., uplift).

In Fig. 4b, we can observe that urban areas with land subsidence below − 2.8 cm/year are along a central east–west region, a central north–south, along the coastal plains in the Gulf of Mexico, northwestern Mexico, and portions of the Baja California Peninsula. One of the most evident areas is the well-documented case of Mexico City (e.g., Solano-Rojas et al. 2020; Chaussard et al. 2021; Fernández-Torres et al. 2022), although some other prominent large areas either with a large subsidence footprint and/or with very fast rates are present (e.g., Sarychikhina et al. 2011; Chaussard et al. 2014).

### Urban localities with land subsidence and land subsidence regions

**Fig. 6:**
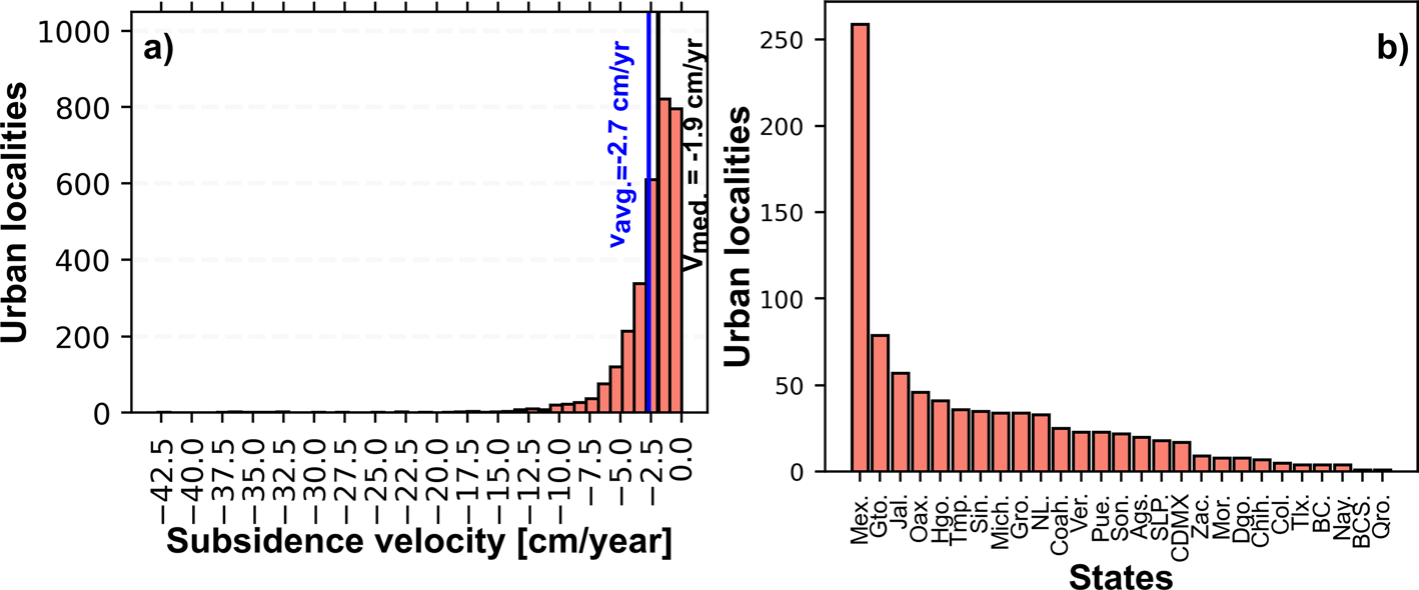
Number of urban localities according to velocities and states. **a** Analysis of maximum subsidence velocity observed in urban localities located inside potential areas with land subsidence in Mexico in 2018–2019. **b** The number of urban localities per state with land subsidence faster than − 2.8 cm/year and slope lower than 5 degrees

The distribution of maximum subsidence velocity per urban localities (presented in Fig. 6a) indicates that 3149 urban localities are subsiding, and 75% of the urban localities have a subsidence velocity faster than − 0.9 cm/year and mean and median velocity values of − 2.7 cm/year and − 1.9 cm/year, respectively (Table 3). Additionally, the fastest land subsidence recorded in an urban area is − 42.8 cm/year at Ciudad Nezahualcoyotl, which is part of the Mexico City Metropolitan Area (Fig. 6a; Table 3). Nevertheless, the maximum land subsidence velocity recorded in Mexico is − 48.3 cm/year in the northeast sector of the Mexico City Metropolitan Area in a not urbanized area within the Ciudad Nezahualcoyotl municipality (Fig. 4a, b).

**Table 3:** Basic statistics of maximum subsidence velocity observed in urban localities located inside of potential areas with land subsidence in Mexico in 2018–2019

Parameter	Value
Count	3.149
Mean	− 2.7 cm/year
Standard deviation	3.4 cm/year
Minimum	− 42.8 cm/year
25%	− 3.3 cm/year
50%	− 1.9 cm/year
75%	− 0.9 cm/year

**Fig. 7:**
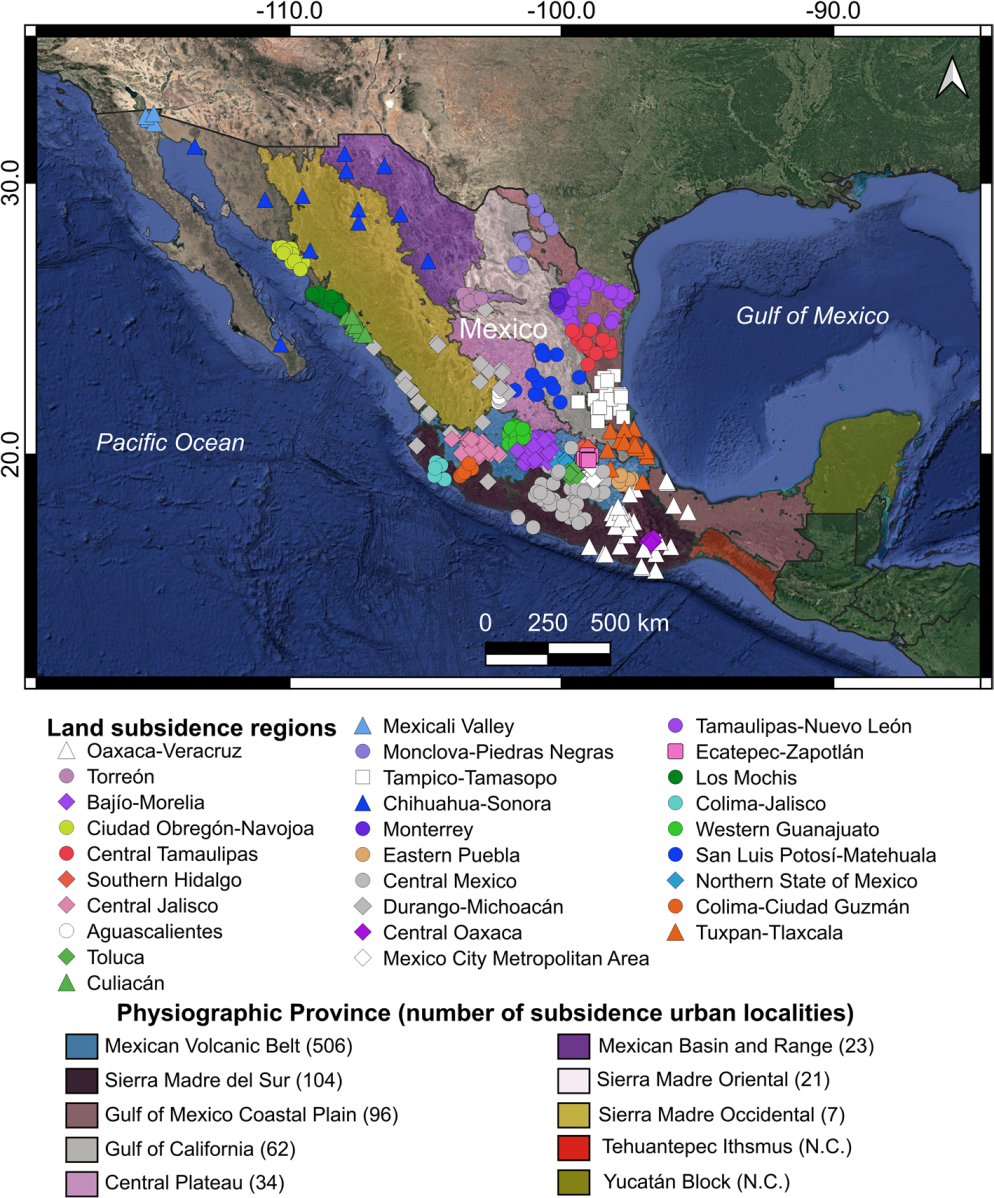
Map of urban localities undergoing land subsidence grouped by regions. Colored polygons are Mexico’s physiographic provinces. N.C., not considered

However, to better compare our results with previous regional observations and to keep low level of noise, we only consider those urban areas with land subsidence velocity faster than − 2.8 cm/year and slope lower than 5 degrees. We detected 853 urban localities within this subsidence velocity and slope thresholds (Figs. 6b, 7; Supplementary file 2). As a result, the State of Mexico (Mex.) has the highest number of urban localities with ground subsidence rates faster than − 2.8 cm/year, representing 30.3% of the total localities detected (Fig. 6b). We divided urban localities undergoing subsidence into three categories: (a) low − 2.8 to − 5 cm/year, (b) intermediate − 5 to − 10 cm/year, and (c) high faster than − 10 cm/year. The highest number of localities and the highest magnitude of subsidence are located within the Mexican Volcanic Belt (MVB; Fig. 7). This geological province has 59.3% of the total number of urban localities undergoing land subsidence. In the Mexican Volcanic Belt, numerous urban areas have developed over flat surfaces, which are, in most cases, the surface expression of endorheic basins or tectonic valleys (grabens and semi-grabens) filled with variable thickness, compressible lacustrine and fluvial sediments interbedded with pyroclasts and lava flows within an extensional tectonic regime (e.g., García-Palomo et al. 2000; Carreón-Freyre and Cerca 2006). The MVB presents compressible deposits that can reach hundreds of meters in thickness which spatially correspond with the higher recorded velocities rates (e.g., Chaussard et al. 2021). In addition, the geomechanical properties of silt and clay compressible deposits indicate low shear strength, variable compression index, and water content that can reach 300%, which promotes the ground subsidence process (e.g., Marsal et al. 2016). The MVB also has the 23.5% of aquifers under stress, in part because this region of Mexico has 90% of irrigated agricultural areas and 76% of Mexico’s population; however, this region only receives 20% of Mexico’s total rain precipitation. (Hernandez 2003). This largely populated region has undergone a rapid development period in the past 40 years and thus groundwater use, and large subsidence velocities.

**Fig. 8:**
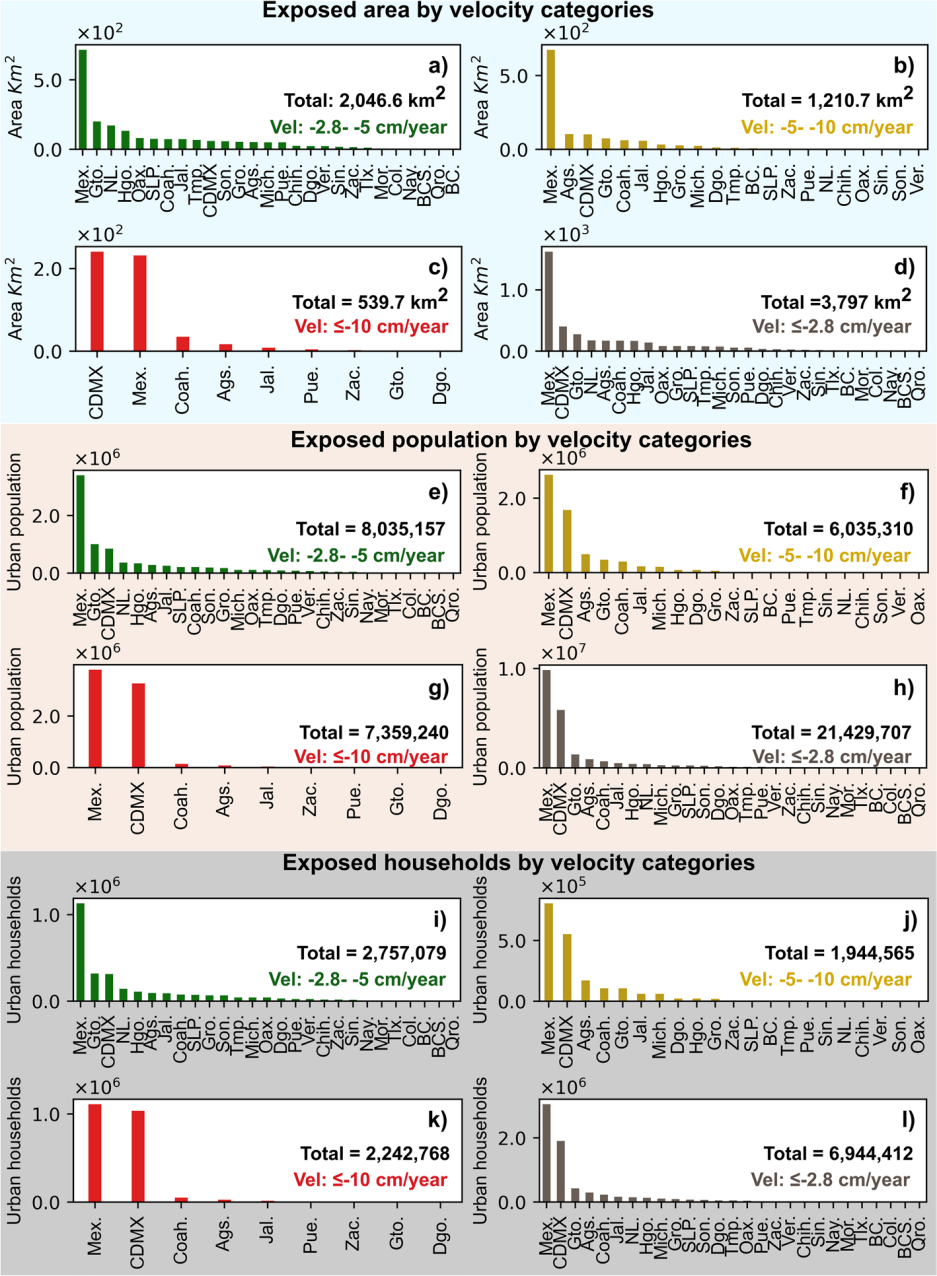
Total area, population and households exposed to land subsidence (≤ − 2.8 cm/year) and slope lower than 5 degrees in Mexico

According to our observations, 27 out of 32 states in Mexico have urban areas with velocities faster or equal to − 2.8 cm/year over slope lower than 5 degrees (Figs. 6b, 8). As a result, the country has a total area of 3797 km^2^ exposed to subsidence (Fig. 8d), where the State of Mexico (Mex.) and Mexico City (CDMX) comprise 53.3% of this subsiding area (Fig. 8d). Due to high population density in this area, 21.4 million people (Fig. 8h) or 17% of the country’s total population in 2020 (INEGI 2020) live in areas exposed to land subsidence faster than − 2.8 cm/year. This population spread throughout 6,944,412 households (Fig. 8l).

The − 2.8 to − 5 cm/year subsidence velocity interval has a total area of 2046.6 km^2^ with a population of 8,035,157, and 2,757,079 households within it (Fig. 8a, e, i respectively). If we consider the subsidence velocity between − 5 and − 10 cm/year, its exposed urban area, population, and households are 1210.7 km^2^, 6,035,310, and 1,944,585, respectively (Fig. 8b, f, j, respectively). The fastest land subsidence interval (≤ − 10 cm/year) has a total area of 539.7 km^2^, exposing 7,359,240 people and 2,242,768 households (Fig. 8c, g, k, respectively) to ground subsidence.

All urban subsiding locations were be grouped into 29 regions (see Fig. 7 and Table 4). Nine of these ground subsidence regions are located within the Mexican Volcanic Belt geologic province (Bajío-Morelia, Southern Hidalgo, Central Jalisco, Toluca, Eastern Puebla, Mexico City Metropolitan Area, Ecatepec-Zapotlán, Western Guanajuato, Northern State of Mexico) and five additional regions are partly located in this physiographic province (Oaxaca-Veracruz, Central Mexico, Durango-Michoacán, Colima-Ciudad Guzman, Tuxpan-Tlaxcala). Consequently, the Mexican Volcanic Belt geologic province has the highest number of land subsidence clusters (Fig. 7). The number of urban localities per land subsidence region ranges from 92 (in Toluca) to 5 (Mexicali Valley) (Table 4). The maximum subsidence velocity found was − 42.8 cm/year located in the Mexico City Metropolitan Area (MCMA). The MCMA land subsidence region has the highest urban area (781.3 km^2^), population (11,141,364), and households (3,501,957) with exposure to land subsidence (see Fig. 7 and Table 4).

**Table 4:** Urban regions with urban land subsidence in Mexico and their total area, population, households, and maximum subsidence velocity

ID	Region name	# Urban localities	Velocity ≤ − 2.8 cm/year			Maximum subsidence velocity cm/year
			Urban area (km^2^)	Population	Households	
0	Oaxaca-Veracruz	29	34.3	40.637	16.244	− 5.3
1	Torreón	18	178.3	784.736	272.697	− 14.3
2	Bajío-Morelia	66	151.6	539.216	196.529	− 12.7
3	Ciudad Obregón-Navojoa	17	52.4	184.823	64.076	− 5.9
4	Central Tamaulipas	13	21.8	26.209	11.415	− 5.5
5	Southern Hidalgo	35	157	392.795	124.986	− 9.9
6	Central Jalisco	47	133.5	411.005	148.692	− 16.5
7	Aguascalientes	20	171.5	865.082	291.471	− 11.1
8	Toluca	92	624.9	2.186.719	662.530	− 11.6
9	Culiacán	12	7.9	18.766	4.967	− 3.9
10	Mexicali Valley	5	7.5	9.762	3.734	− 9.1
11	Monclova-Piedras Negras	12	24	17.971	6.847	− 5
12	Tampico-Tamasopo	19	32.1	70.219	26.251	− 6.4
13	Chihuahua-Sonora	12	35.9	64.539	24.213	− 7
14	Monterrey	18	115.1	281.102	108.056	− 3.9
15	Eastern Puebla	17	51.9	82.881	25.026	− 14.3
16	Central Mexico	58	117.3	294.503	110.672	− 7.9
17	Durango-Michoacán	27	58.1	249.477	90.655	− 19.3
18	Central Oaxaca	24	56.9	82.652	31.440	− 4.8
19	Mexico City Metropolitan Area	65	781.3	11.141.364	3.501.957	− 42.8
20	Tamaulipas-Nuevo León	31	96.3	130.892	55.710	− 6.7
21	Ecatepec-Zapotlán	78	321.2	1.846.946	649.590	− 12.9
22	Los Mochis	19	8.3	23.286	7.166	− 5.2
23	Colima-Jalisco	7	2.8	6.802	2.683	− 4.2
24	Western Guanajuato	32	151.5	890.096	265.169	− 9.2
25	San Luis Potosí-Matehuala	13	73.5	214.330	73.154	− 6.5
26	Northern State of Mexico	41	296.1	469.884	134.124	− 6.6
27	Colima-Ciudad Guzmán	10	17.3	66.635	22.323	− 7.6
28	Tuxpan-Tlaxcala	16	16.4	36.378	12.035	− 4.3

### Relationship between land subsidence and conditional factors

**Fig. 9:**
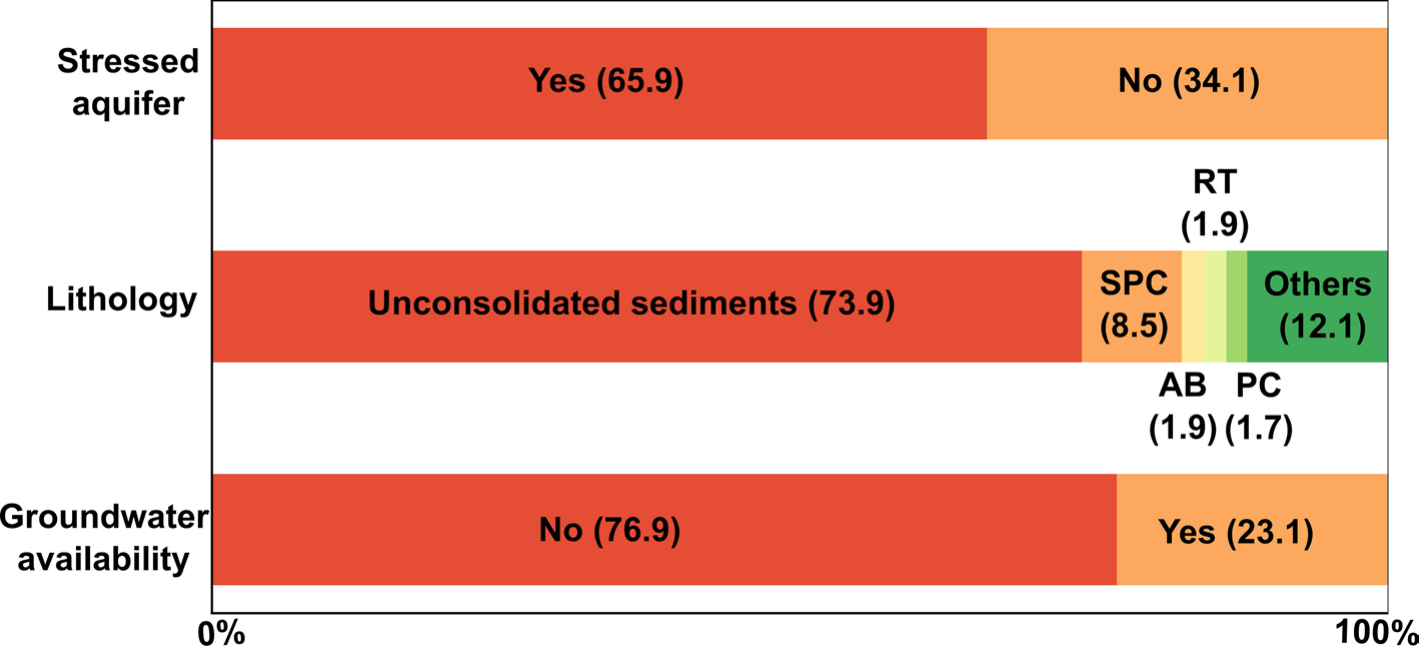
Spatial distribution of land subsidence conditional factors. SPC, sandstone-polygenic conglomerate; AB, andesite-basalt; RT, rhyolite tuff; PC, polygenic conglomerate; other lithologies (e.g., andesitic tuff, limestone, shale, among others)

We evaluated the relationship between land subsidence and three variables that generate favorable conditions for land subsidence development associated with groundwater extraction. These variables are lithology, groundwater availability, and aquifer stress (Fig. 9).

To evaluate groundwater depletion’s influence over land subsidence in urban areas, we considered groundwater availability and whether the aquifers were stressed. These variables showed that 76.9% and 65.9% of the urban areas with velocities faster than − 2.8 cm/year and slope lower than 5 degrees have no groundwater availability and an underlying stressed aquifer, respectively (Fig. 9). As a result, land subsidence generally occurred in water-stressed basins, where groundwater withdrawal rate is higher than the natural recharge.

Our results show that 73.9% (~ 2919.9 km^2^) of the urban areas with land subsidence are developed over unconsolidated sediments, 8.5% of the urban areas developed over sandstones-polygenic conglomerate (SPC), 1.9% are over andesitic-basalt (AB), 1.9% in rhyolitic tuff (RT), 1.7% are over polygenic conglomerate, and 12.1% over other lithologies (e.g., andesitic tuff, shale, limestones, among others). These results indicate that most exposed urban areas are located over unconsolidated sediments, although we also notice a large lithological heterogeneity (see Fig. 9).

### InSAR and GPS comparison

**Fig. 10:**
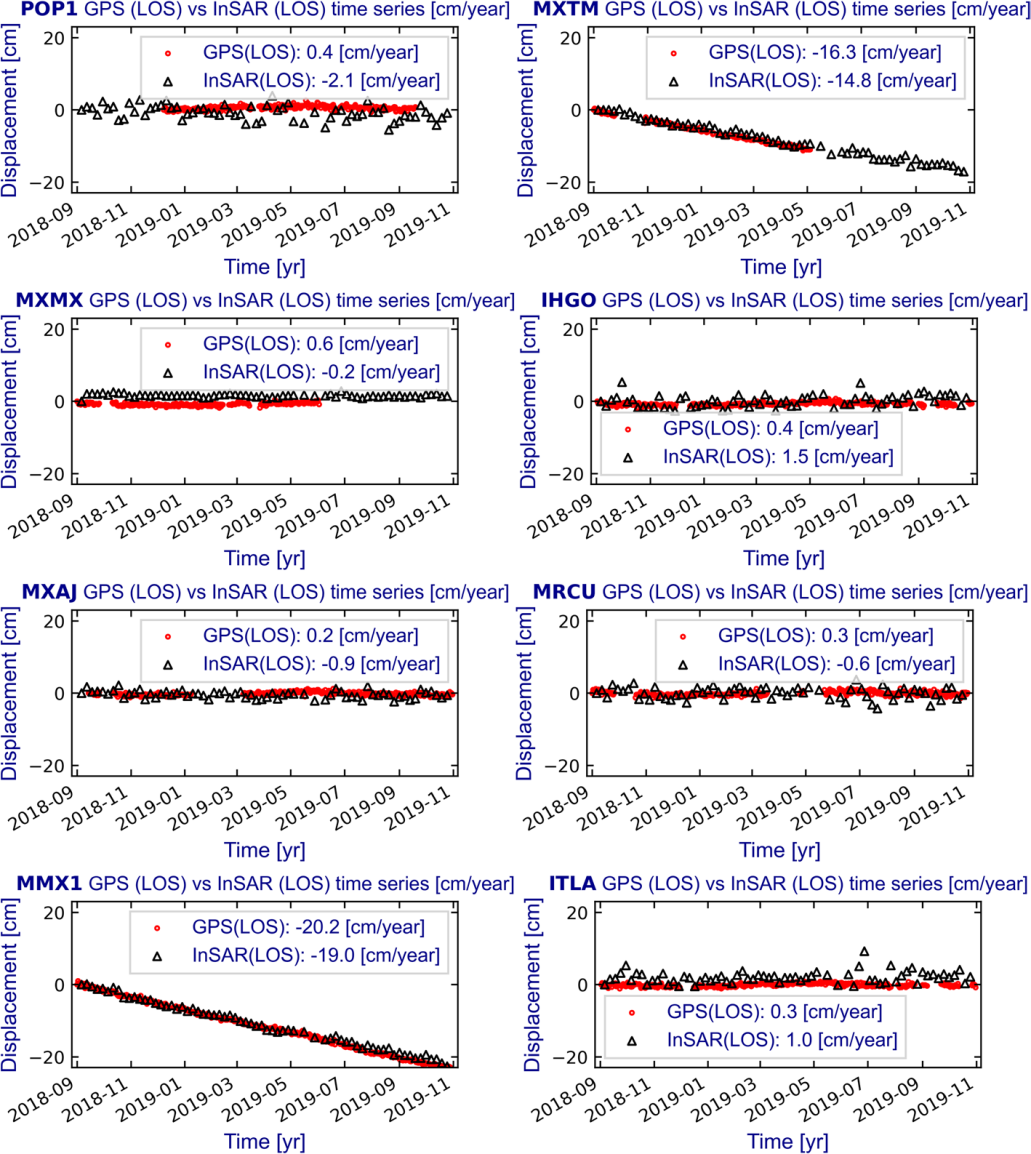
Comparison between the InSAR (LOS; black triangles) and the LOS-projected GPS (red dots) surface deformation time series for 14 out of 100 GPS stations identified in Fig. 5

**Fig. 11:**
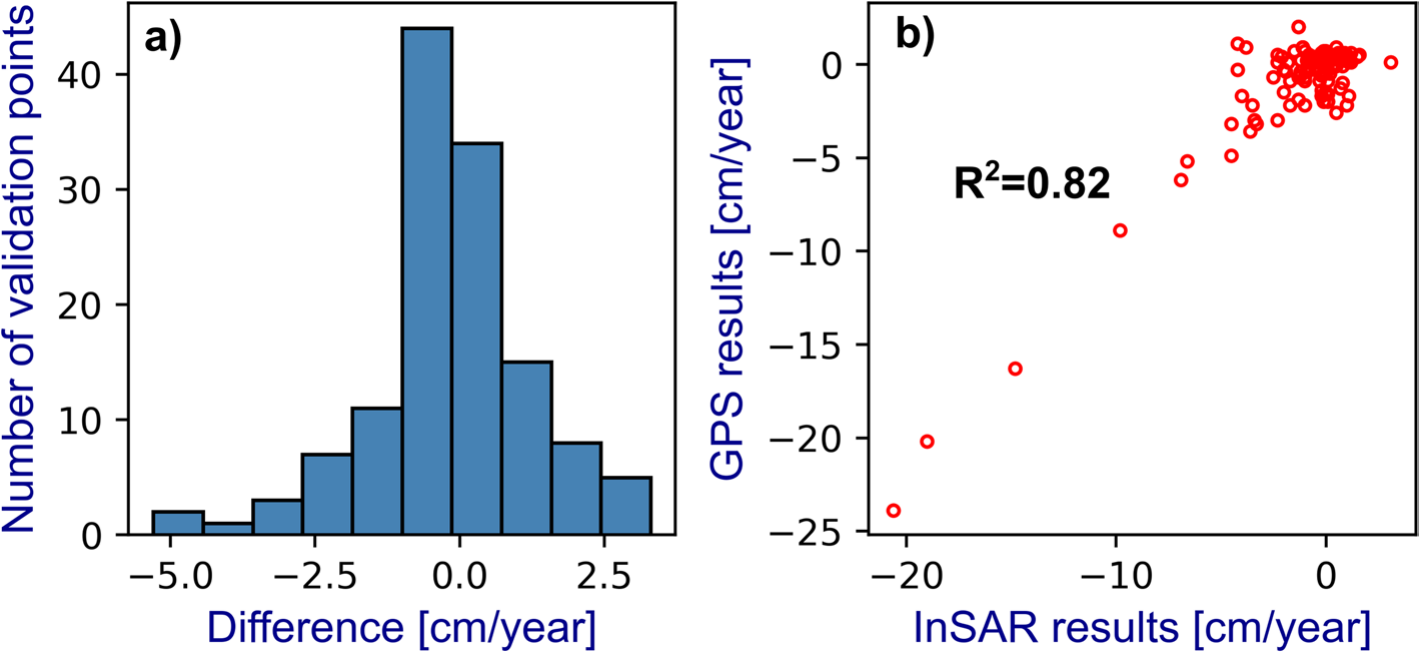
Statistics of the differences between InSAR and GPS velocities. **a** Histogram of the velocity’s differences between InSAR and GPS. **b** Scatter plot with the correlation among InSAR and GPS velocities

InSAR and GPS time series velocities (LOS) comparisons were carried out to assess the accuracy of our InSAR results. These comparisons are shown in Fig. 10, and the Supplementary material (Supplementary file 4). In this analysis, we used 100 permanent GPS stations located on high coherence pixels (temporal coherence > 0.7) (Figs. 5, 10; Supplementary file 3, Supplementary file 4, and Supplementary file 5). Furthermore, due to the overlapping nature between relative orbits of Sentinel-1 (Figs. 3, 11) those 100 GPS locations provide 130 calibration points on the InSAR velocity maps.

InSAR time series accuracy was obtained by computing the differences between InSAR and GPS in LOS velocities (Fig. 11a; Table 5). Our results show that the differences are very close to a normal distribution where mean and median values are − 1 mm/year and − 2 mm/year, respectively. Thus, most of the velocity differences are near 0 cm/year (Table 5). We also found the correlation between GPS (LOS) and InSAR (LOS) velocities shows a coefficient (*R*^2^) of 0.82 (Fig. 11b), indicating a very high correlation. Besides, using the three GPS components to project into LOS to assess the accuracy of LOS InSAR measurements, as has been previously used elsewhere (e.g., Manunta et al. 2019). Obtaining − 1 mm/year in the mean difference velocities suggests that the LOS InSAR measurements are accurate, and GPS horizontal velocity components are negligible; thus, the projection of InSAR LOS as a vertical component is a valid assumption.

**Table 5:** Basic statistics of the differences between InSAR and GPS velocities

Parameter	Value
Count	130
Mean (cm/year)	− 0.1
Std. (cm/year)	1.4
Min. (cm/year)	− 5.3
25% (cm/year)	− 0.8
50% (cm/year)	− 0.2
75% (cm/year)	0.6
Max. (cm/year)	3.3

## Discussion

We performed a country-wide assessment of areas, population, and households exposed to land subsidence using InSAR-SBAS in Mexico and an extensive comparison between InSAR and GPS velocities to calibrate our results. Our primary objective was to identify urban subsidence; thus, this assessment did not account for all agricultural areas that also undergo subsidence. If we consider a threshold detection limit of − 2.8 cm/year and slope lower than 5 degrees, 3797 km^2^ of urbanized areas are affected by land subsidence, and consequently, 21.4 million people and 6.9 million households are exposed to significant land subsidence. In context, more than 15.7% out of 24,227 km^2^ urban AGEB area of Mexico probably exceed the tolerable limits of settlement that a civil structure may undergo under current building codes (e.g., Gaceta Oficial de la Ciudad de México 2017). This result indicates that a considerable proportion of the critical urban infrastructure (e.g., roads, hospitals, schools, surface and underground transportation and underground hydraulic lines, etc.), as well as households, are affected by land subsidence, and therefore a sharp decrease in their useful life is expected with long-term financial consequences. However, building damage severity due to ground subsidence not only depends on a high subsidence rate, but also on the differential settlement, angular distortion, construction quality, foundations type, and other underlying lithological and geomechanical properties (e.g., Peduto et al. 2017).

All detected urban areas with ground subsidence faster than − 2.8 cm/year were grouped into 29 land subsidence regions, and these are in the central east–west trending Mexican Volcanic Belt, the central highland plateau, along the coastal plains in the Gulf of Mexico, the northwestern Mexico coastal plains, and in some portions of the Baja California Peninsula (Figs. 4, 7 and Table 4). The conditional factors for this spatial pattern are high population density, the prevalence of unconsolidated sediment, and underlying groundwater-stressed basins (Fig. 1). These results bear strong similarities with the Potential Global subsidence map (Herrera-García et al. 2021). However, this map only shows high and very high land subsidence potential in the northwestern portion of the Gulf of Mexico and some small areas inside the central portion of Mexico. Possible reasons for differences between our results and the Herrera-García et al. (2021) work is their worldwide scope and, consequently, the inherently lower spatial resolution of global datasets used. Nevertheless, there is a general good agreement.

Our land subsidence magnitude observations show that several of the Mexican urban localities fall within the highest ground subsidence rates registered around the world (e.g., Galloway and Burbey 2011) such as Ciudad Nezahualcóyotl, part of the Mexico City metropolitan area [− 42.8 cm/year], Chaparrosa, Zacatecas [− 19.3 cm/year], Jocotepec, Jalisco [− 16.5 cm/year], La Partida, Coahuila [− 14.3 cm/year], La Purisima, Puebla [− 14.3 cm/year], Santa Rosa, Guanajuato [− 12.7 cm/year] (Supplementary file 1).

Spatial pattern and subsidence rates evolution can be evaluated by comparing our results with previously reported subsidence areas (e.g., the east–west central Mexico and Cerro Prieto regions; see Supplementary file 6 for details) and evaluation of the spatial distribution and velocity rate consistency throughout a longer time span (Supplementary file 1). For most of all the previously recognized subsiding areas, we observe very similar magnitudes and overall spatial distribution (Supplementary file 6). The subsidence rates and spatial consistency indicate that most of the localities present a linear subsidence behavior, and their velocities are largely unchanged. The only exception is the city of Querétaro, where changes in the water sources have made possible a 3 × reduction after 2011, where subsidence rates dropped from ~ − 5 cm/year to the current rates of ~ − 1.5 cm/year. This subsidence deceleration implies that water management modifications can indeed mitigate land subsidence due to groundwater extraction (e.g., Morishita 2021). On the other hand, we also record some differences (although far fewer locations) between our results and previous reported maximum average ground subsidence rates (e.g., Ahucatlán, Ciudad Guzmán, Mexicali; Supplementary file 6) that may be caused by multiple factors. Other than a simple increase in the extraction rate that triggers changes in subsidence velocity or spatial distribution, we compared the InSAR time series of prominent subsidence areas with longer time series (3.5 years), obtaining similar velocity values and spatial distribution (see Supplementary file 1); consequently, the September 2018–October 2019 present consistency when comparing with longer time series.

We have also investigated the possible conditional factors for all ground subsidence and found that urban areas with subsidence rates faster than − 2.8 cm/year were preferably developed over unconsolidated sediments (73.9%), without excess groundwater availability (76.9%), and over an underlying stressed aquifer (65.9%). These unconsolidated sediments are primarily deposited over coastal plains, or over structurally controlled or endorheic lacustrine basins where a low relief is dominant. These sedimentary environments favor ground subsidence because of the compressible mechanical behavior of the infills and where these unconsolidated materials are more prone to compaction due to their decreasing pore water pressure (e.g., Carrillo 1948). Water pressure decreasing over deformable unconsolidated deposits is, by far, the main trigger of ground lowering (e.g., Motagh et al. 2017); consequently, non-groundwater availability and stressed spatial distribution, as we have documented, exhibit a very close relationship with urban areas undergoing land subsidence. Our land subsidence assessment in Mexico and the conditional factors are very well-aligned and (at least regionally) corroborate previous large-scale subsidence potential distribution (e.g., Herrera-García et al. 2021).

The precision of our InSAR-SBAS analysis was assessed through an extensive comparison with 100 cGPS stations throughout Mexico. The mean velocity differences of − 1 mm/year were obtained when we compared the GPS (LOS) and InSAR (LOS) time series (Table 5); therefore, our results show a millimetric accuracy level and is similar to results than analogous surveys (e.g., Manunta et al. 2019). Three main factors benefit the higher obtained accuracy:

1. Target urban areas and a large portion of GPS stations are in low relief areas that retain long-term spatial coherence and are thus less susceptible to topography removal and phase unwrapping errors, (e.g., Morishita 2021).
2. Pixels with temporal coherence lower than 0.7 were masked from our average velocity maps and time series (e.g., Pepe and Lanari 2006).
3. The Sentinel-1 SAR free data policy and small spatial and temporal baseline (e.g., Torres et al. 2012) allow InSAR analysis at the country-scale level with high temporal resolution.

This study constitutes the most extensive land subsidence assessment that has been performed in Mexico to date; as a result, we detected well over one order of magnitude more localities than all preceding studies (see Supplementary file 2). Perhaps most relevant is the significant increase in the number of detected localities with ground subsidence.

## Conclusion

Detection of urban localities with land subsidence is a necessary step to an adequate and sustainable groundwater and land-use management. This InSAR-SBAS based on Sentinel-1 analysis is the first systematic nation-wide inventory of urban areas, population, and household exposed to land subsidence. We detected 853 urban localities exposed to land subsidence (faster than − 2.8 cm/year and slope lower than 5 degrees) which were clustered into 29 land subsidence regions. These subsiding urban centers cover of 3797 km^2^, or about 15.7% of the total urban area of Mexico. Moreover, land subsidence areas expose 21.4 million people and 6.9 million households.

Our results also show that detected land subsidence velocities have the same rate and spatial distribution that have been previously reported smaller coverage studies in central Mexico (e.g., Chaussard et al. 2014, indicating that any subsidence mitigation efforts have been insufficient) except for the city of Querétaro, where subsidence rates have decreased from ~ − 5 to ~ − 1.5 cm/year due to changes in water management (Castellazzi et al. 2021). The obtained urban subsidence rates are among the highest in the world (e.g., Galloway and Burbey 2011), and we also found that it is spatially controlled by groundwater and geological setting conditions. Regarding groundwater, we found that (65.9%) and (76.9%) of the urban area with land subsidence is over-stressed aquifers and no groundwater availability, respectively (Fig. 9). In the case of geological conditions, our results show that (73.9%) of the total urban area with land subsidence faster than − 2.8 cm/year are on unconsolidated sediments (mainly lacustrine and alluvial deposits; Fig. 9). In other words, around 7 of each 10 km^2^ of the urban area with land subsidence occurs where the water table considerably decreases and with highly compressible deposits, which in some cases can reach several hundreds of meters. The obtained urban land subsidence panorama may be a consequence of the no planning for city growth that Mexico has experienced in the last 40 years because of accelerated population growth (INEGI 2020), causing an increase in the probability of groundwater contamination and damages in building and infrastructure (Hernández-Espriú et al. 2014; Solano-Rojas et al. 2020). In addition, if no effective strategies are applied, the current land subsidence status can continue or even can be worst in the following decades, considering the possible growth in groundwater dependency because of climate change scenarios which forecast a rise in intensity and duration of drought (IPCC 2021).

The results of this research have local and general impacts in terms of land subsidence. At the local scale, urban land subsidence detection can be helpful to Civil Protection authorities and decision-makers to improve urban planning, water management strategies, assessment and mitigation of risk associated and subsidence, and to identify urban are where field measurements and in-depth research is needed. Moreover, this study has covered ~ 85% of the Mexican territory, identifying urban areas undergoing ground subsidence that were not previously detected and documented. The spatial relationships between urban land subsidence and triggering factors (i.e., lithology, groundwater stress, and groundwater availability) were assessed and confirmed results of previous works (e.g., Herrera-Garcia et al. 2021).

### Supplementary information

*Supplementary file 1* compares September 2018–October 2019, and January 2018–June 2021 InSAR time series in urban areas with prominent land subsidence rates*. Supplementary file 2* contains detailed results and other ancillary information used in the analysis of 853 urban localities in Mexico that undergo land subsidence. *Supplementary file 3* has the time overlap between GPS and InSAR time series. *Supplementary file 4* shows the comparative analysis between 130 InSAR and GPS (LOS) time series. *Supplementary file 5* presents the location and geographic coordinates of the GPS stations used in this study. *Supplementary file 6* has the subsidence velocity comparison in previous reported areas.

### Supplementary Information

Below is the link to the electronic supplementary material.

Supplementary file1 (DOCX 6590 KB)

Supplementary file2 (XLSX 134 KB)

Supplementary file3 (XLSX 12 KB)

Supplementary file4 (DOCX 52694 KB)

Supplementary file5 (XLSX 13 KB)

Supplementary file6 (DOCX 21 KB)
