# Supplementary material for: Country-scale assessment of urban areas, population, and households exposed to land subsidence using Sentinel-1 InSAR, and GPS time series
Source: Nat Hazards (Dordr). 2023 Oct 29;120(2):1577–601. doi: 10.1007/s11069-023-06259-5 (PMC10824816; doi:10.1007/s11069-023-06259-5)
Supplement: Supplementary file 1 — Supplementary file1 (DOCX 6590 KB) [file 11069_2023_6259_MOESM1_ESM.docx]

**Country-scale assessment of urban areas, population, and households exposed to land subsidence using Sentinel-1 InSAR and GPS time series**

Enrique Antonio Fernández-Torres^a,b^, Enrique Cabral-Cano^b^, Darío Solano-Rojas^c^, Luis Salazar-Tlaczani^b^, Josue Gárcia-Venegas^c^, Bertha Marquez-Azúa^d^, Shannon Graham^e^, Katia Michelle Villarnobo-Gonzalez^f^.

^a^ Posgrado en Ciencias de la Tierra, Universidad Nacional Autónoma de México. Ciudad Universitaria, Coyoacán, CDMX, 04510, México

^b^ Departamento de Geomagnetismo y Exploración, Instituto de Geofísica, Universidad Nacional Autónoma de México. Ciudad Universitaria, Coyoacán, CDMX, 04510, México

^c^ División de Ingeniería en Ciencias de la Tierra, Facultad de Ingeniería, Universidad Nacional Autónoma de México. Ciudad Universitaria, Coyoacán, CDMX, 04510, México

^d^ Centro de Estudios Estratégicos para el Desarrollo, Universidad de Guadalajara, Tomás V. Gómez 121, Ladrón de Guevara, Guadalajara, Jalisco 44100, México

^e^ The College of New Jersey Physics Department, 2000 Pennington Rd. Ewing, NJ 08628, USA

^f^Departamento de Física, Facultad de Ciencias, Universidad Nacional Autónoma de México. Ciudad Universitaria, Coyoacán, CDMX, 04510, México

Correspondence to:

Enrique Antonio Fernandez-Torres

enrique.30065@gmail.com

**Supplementary material**


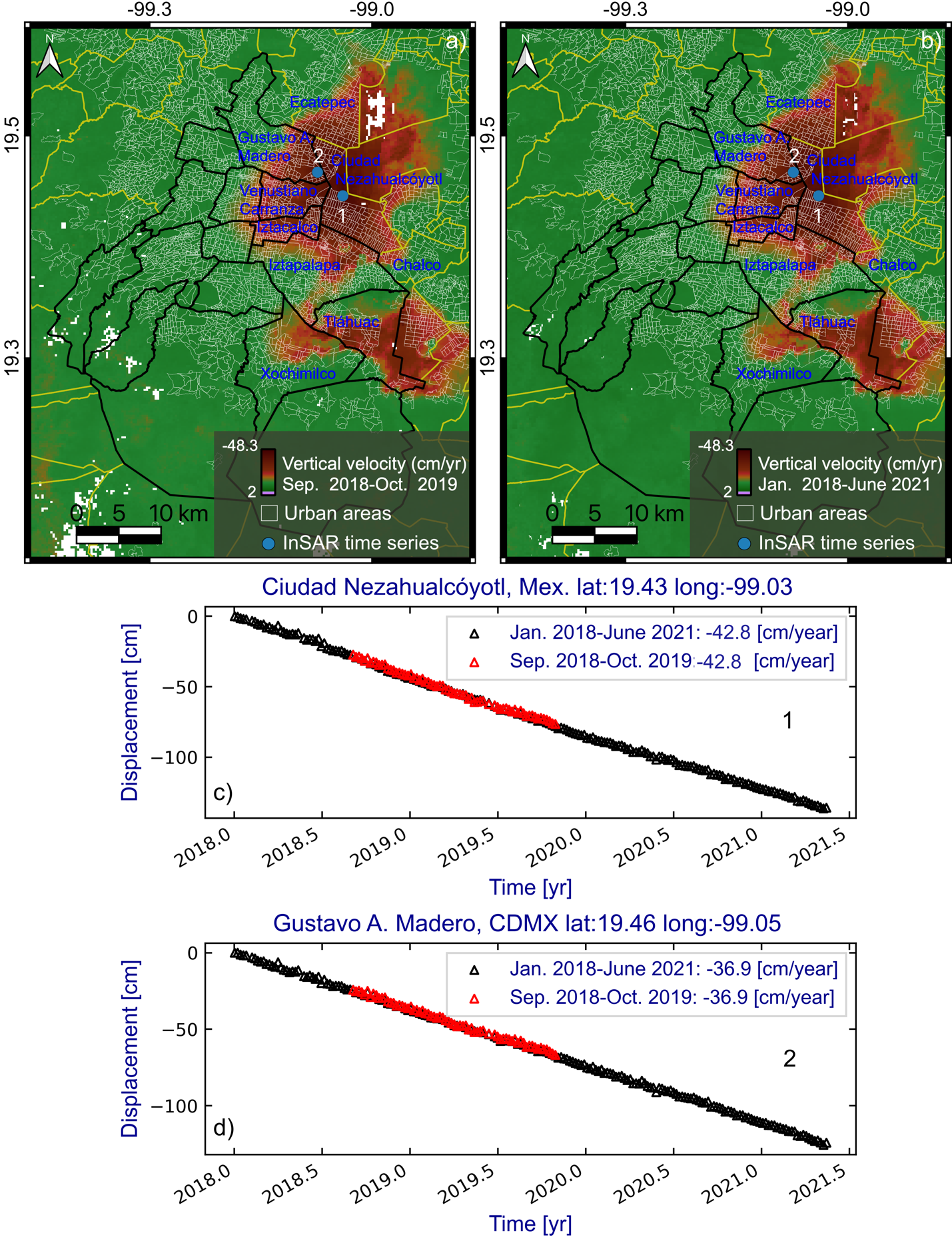


Figure *S1*: Comparison between Jan. 2018-June 2021 and Sep. 2018-Oct 2019 vertical velocities in the Mexico City Metropolitan area. a) Vertical velocity map of Mexico City Metropolitan area Jan. 2018-June 2019. b) Vertical velocity map of Mexico City Metropolitan area Sep. 2018-Oct 2021. c) Comparison between Jan. 2018-June 2021 and Sep. 2018-Oct 2019 vertical velocities InSAR time series at Ciudad Netzahualcóyotl. d) Comparison between Jan. 2018-June 2021 and Sep. 2018-Oct 2019 vertical velocities InSAR time series at Gustavo A. Madero. Black and yellow polygons represent the Mexico City and State of Mexico municipalities, respectively


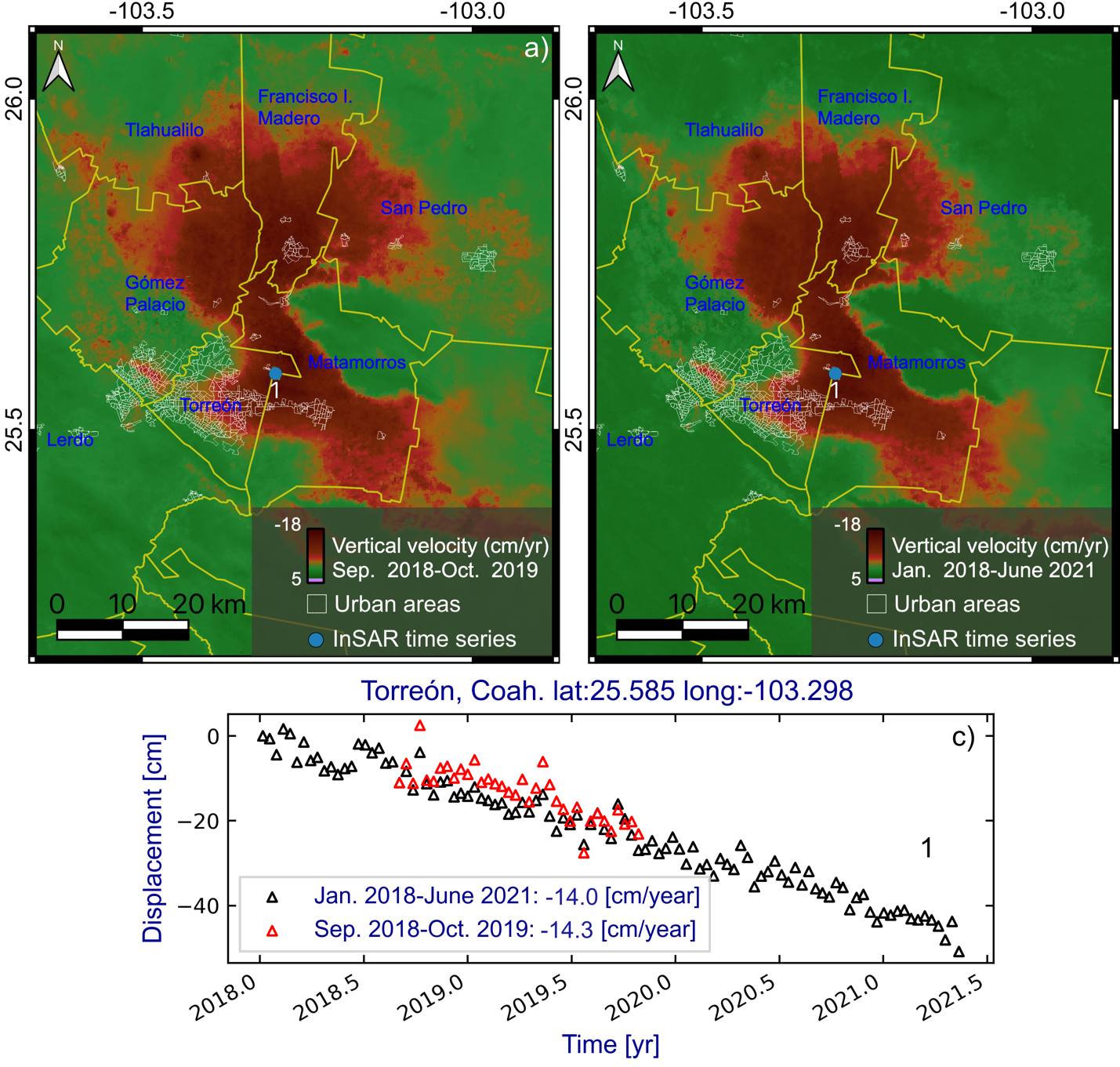


Figure *S2*: Comparison between Jan. 2018-June 2021 and Sep. 2018-Oct 2019 vertical velocities in Torreón area and surrounding. a) Vertical velocity map of Torreón Jan. 2018-June 2019. b) Vertical velocity map of Torreón Sep. 2018-Oct 2021. c) Comparison between Jan. 2018-June 2021 and Sep. 2018-Oct 2019 vertical velocities InSAR time series at La Partida, Torreón. Yellow polygons represent municipalities


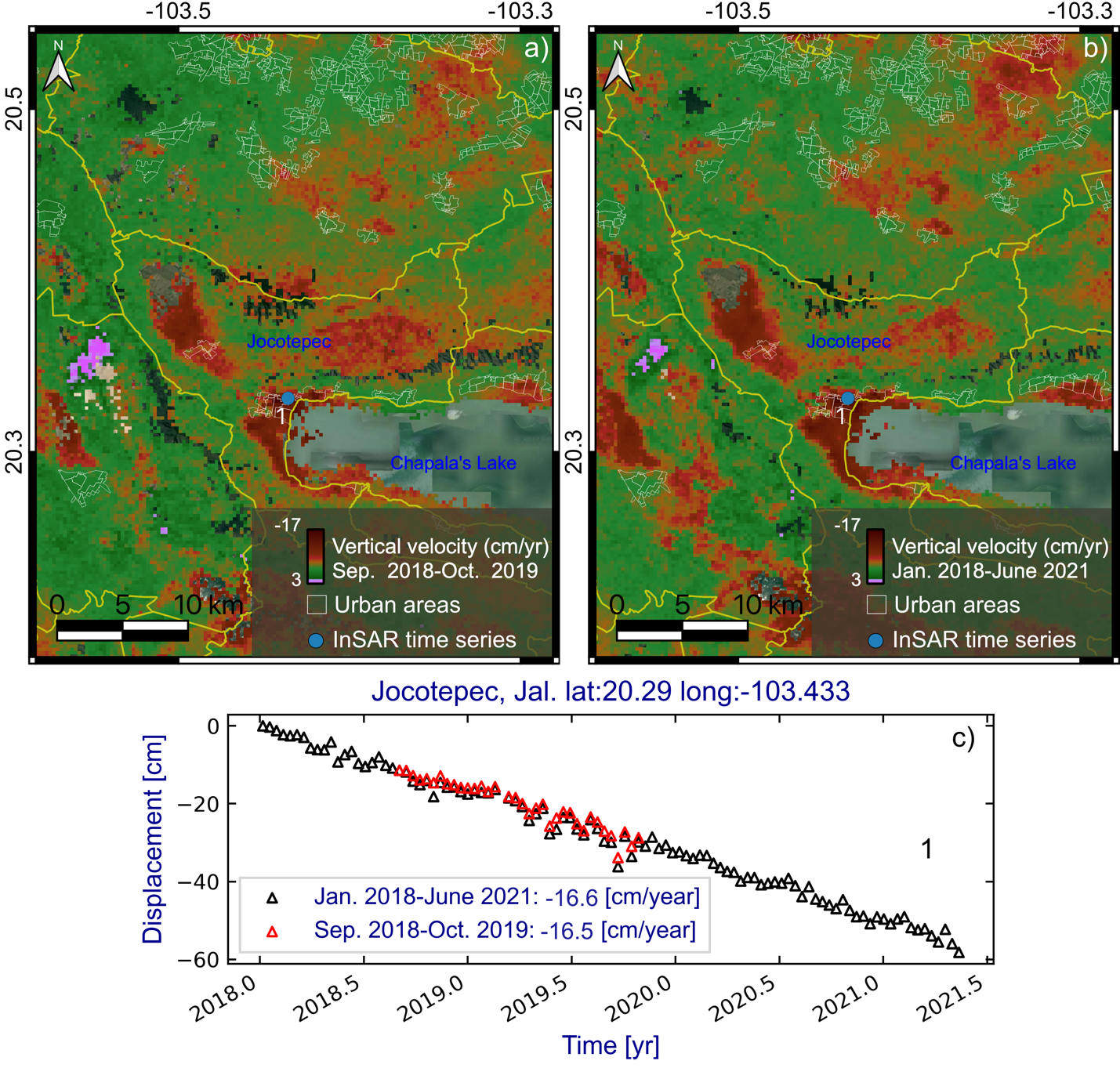


Figure *S3:* Comparison between Jan. 2018-June 2021 and Sep. 2018-Oct 2019 vertical velocities in Jocotepec area and surrounding. a) Vertical velocity map of Jocotepec Jan. 2018-June 2019. b) Vertical velocity map of Jocotepec Sep. 2018-Oct 2021. c) Comparison between Jan. 2018-June 2021 and Sep. 2018-Oct 2019 vertical velocities InSAR time series at Jocotepec, Jalisco. Yellow polygons represent municipalities


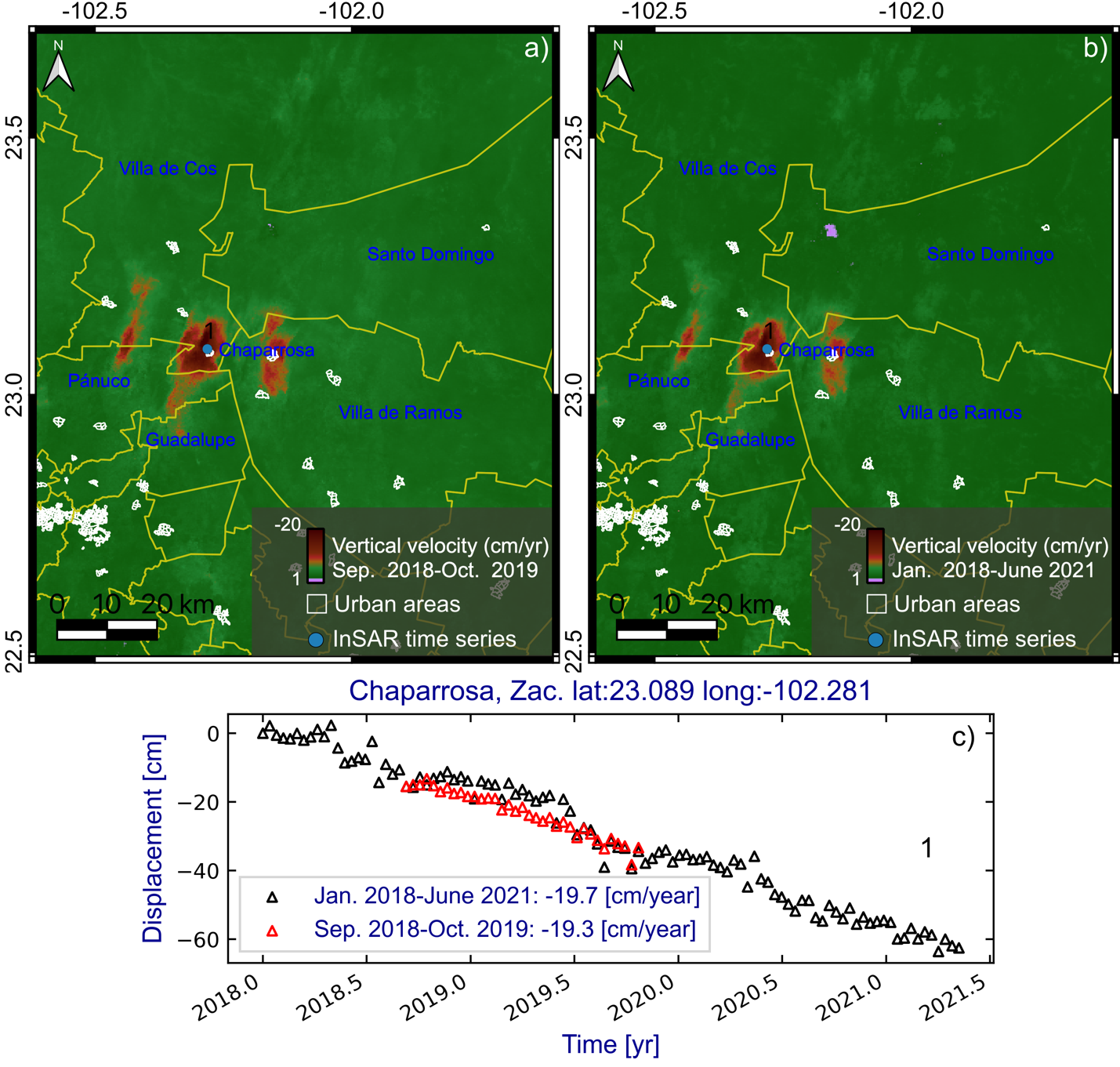


Figure *S4:* Comparison between Jan. 2018-June 2021 and Sep. 2018-Oct 2019 vertical velocities in Chaparrosa area and surrounding. a) Vertical velocity map of Chaparrosa Jan. 2018-June 2019. b) Vertical velocity map of Chaparrosa Sep. 2018-Oct 2021. c) Comparison between Jan. 2018-June 2021 and Sep. 2018-Oct 2019 vertical velocities InSAR time series at Chaparrosa, Zacatecas. Yellow polygons represent municipalities
