# Supplementary material for: Country-scale assessment of urban areas, population, and households exposed to land subsidence using Sentinel-1 InSAR, and GPS time series
Source: Nat Hazards (Dordr). 2023 Oct 29;120(2):1577–601. doi: 10.1007/s11069-023-06259-5 (PMC10824816; doi:10.1007/s11069-023-06259-5)
Supplement: Supplementary file 4 — Supplementary file4 (DOCX 52694 KB) [file 11069_2023_6259_MOESM4_ESM.docx]

**Country-scale assessment of urban areas, population, and households exposed to land subsidence using Sentinel-1 InSAR and GPS time series**

Enrique Antonio Fernández-Torres^a,b^, Enrique Cabral-Cano^b^, Darío Solano-Rojas^c^, Luis Salazar-Tlaczani^b^, Josue Gárcia-Venegas^c^, Bertha Marquez-Azúa^d^, Shannon Graham^e^, Katia Michelle Villarnobo-Gonzalez^f^.

^a^ Posgrado en Ciencias de la Tierra, Universidad Nacional Autónoma de México. Ciudad Universitaria, Coyoacán, CDMX, 04510, México

^b^ Departamento de Geomagnetismo y Exploración, Instituto de Geofísica, Universidad Nacional Autónoma de México. Ciudad Universitaria, Coyoacán, CDMX, 04510, México

^c^ División de Ingeniería en Ciencias de la Tierra, Facultad de Ingeniería, Universidad Nacional Autónoma de México. Ciudad Universitaria, Coyoacán, CDMX, 04510, México

^d^ Centro de Estudios Estratégicos para el Desarrollo, Universidad de Guadalajara, Tomás V. Gómez 121, Ladrón de Guevara, Guadalajara, Jalisco 44100, México

^e^ The College of New Jersey Physics Department, 2000 Pennington Rd. Ewing, NJ 08628, USA

^f^Departamento de Física, Facultad de Ciencias, Universidad Nacional Autónoma de México. Ciudad Universitaria, Coyoacán, CDMX, 04510, México

Correspondence to:

Enrique Antonio Fernandez-Torres

enrique.30065@gmail.com

**Supplementary material**





Figure *S1:* Comparison between the InSAR (LOS; black triangles) and the LOS-projected GPS (red dots) surface deformation time series for 8 out of 100 GPS stations identified in Fig. 5





Figure *S2:* Comparison between the InSAR (LOS; black triangles) and the LOS-projected GPS (red dots) surface deformation time series for 8 out of 100 GPS stations identified in Fig. 5





Figure *S3:* Comparison between the InSAR (LOS; black triangles) and the LOS-projected GPS (red dots) surface deformation time series for 8 out of 100 GPS stations identified in Fig. 5





Figure *S4:* Comparison between the InSAR (LOS; black triangles) and the LOS-projected GPS (red dots) surface deformation time series for 8 out of 100 GPS stations identified in Fig. 5





Figure *S5:* Comparison between the InSAR (LOS; black triangles) and the LOS-projected GPS (red dots) surface deformation time series for 8 out of 100 GPS stations identified in Fig. 5





Figure *S6:* Comparison between the InSAR (LOS; black triangles) and the LOS-projected GPS (red dots) surface deformation time series for 8 out of 100 GPS stations identified in Fig. 5





Figure *S7:* Comparison between the InSAR (LOS; black triangles) and the LOS-projected GPS (red dots) surface deformation time series for 8 out of 100 GPS stations identified in Fig. 5





Figure *S8:* Comparison between the InSAR (LOS; black triangles) and the LOS-projected GPS (red dots) surface deformation time series for 8 out of 100 GPS stations identified in Fig. 5





Figure *S9:* Comparison between the InSAR (LOS; black triangles) and the LOS-projected GPS (red dots) surface deformation time series for 8 out of 100 GPS stations identified in Fig. 5





Figure *S10:* Comparison between the InSAR (LOS; black triangles) and the LOS-projected GPS (red dots) surface deformation time series for 8 out of 100 GPS stations identified in Fig. 5





Figure *S11:* Comparison between the InSAR (LOS; black triangles) and the LOS-projected GPS (red dots) surface deformation time series for 8 out of 100 GPS stations identified in Fig. 5





Figure *S12:* Comparison between the InSAR (LOS; black triangles) and the LOS-projected GPS (red dots) surface deformation time series for 8 out of 100 GPS stations identified in Fig. 5





Figure *S13*: Comparison between the InSAR (LOS; black triangles) and the LOS-projected GPS (red dots) surface deformation time series for 8 out of 100 GP S stations identified in Fig. 5





Figure *S14:* Comparison between the InSAR (LOS; black triangles) and the LOS-projected GPS (red dots) surface deformation time series for 8 out of 100 GP S stations identified in Fig. 5





Figure *S15:* Comparison between the InSAR (LOS; black triangles) and the LOS-projected GPS (red dots) surface deformation time series for 8 out of 100 GP S stations identified in Fig. 5





Figure *S16:* Comparison between the InSAR (LOS; black triangles) and the LOS-projected GPS (red dots) surface deformation time series for 8 out of 100 GP S stations identified in Fig. 5





Figure *S17:* Comparison between the InSAR (LOS; black triangles) and the LOS-projected GPS (red dots) surface deformation time series for 8 out of 100 GP S stations identified in Fig. 5
