# Supplementary material for: Country-scale assessment of urban areas, population, and households exposed to land subsidence using Sentinel-1 InSAR, and GPS time series
Source: Nat Hazards (Dordr). 2023 Oct 29;120(2):1577–601. doi: 10.1007/s11069-023-06259-5 (PMC10824816; doi:10.1007/s11069-023-06259-5)
Supplement: Supplementary file 6 — Supplementary file6 (DOCX 21 KB) [file 11069_2023_6259_MOESM6_ESM.docx]

**Country-scale assessment of urban areas, population, and households exposed to land subsidence using Sentinel-1 InSAR and GPS time series**

Enrique Antonio Fernández-Torres^a,b^, Enrique Cabral-Cano^b^, Darío Solano-Rojas^c^, Luis Salazar-Tlaczani^b^, Josue Gárcia-Venegas^c^, Bertha Marquez-Azúa^d^, Shannon Graham^e^, Katia Michelle Villarnobo-Gonzalez^f^.

^a^ Posgrado en Ciencias de la Tierra, Universidad Nacional Autónoma de México. Ciudad Universitaria, Coyoacán, CDMX, 04510, México

^b^ Departamento de Geomagnetismo y Exploración, Instituto de Geofísica, Universidad Nacional Autónoma de México. Ciudad Universitaria, Coyoacán, CDMX, 04510, México

^c^ División de Ingeniería en Ciencias de la Tierra, Facultad de Ingeniería, Universidad Nacional Autónoma de México. Ciudad Universitaria, Coyoacán, CDMX, 04510, México

^d^ Centro de Estudios Estratégicos para el Desarrollo, Universidad de Guadalajara, Tomás V. Gómez 121, Ladrón de Guevara, Guadalajara, Jalisco 44100, México

^e^ The College of New Jersey Physics Department, 2000 Pennington Rd. Ewing, NJ 08628, USA

^f^Departamento de Física, Facultad de Ciencias, Universidad Nacional Autónoma de México. Ciudad Universitaria, Coyoacán, CDMX, 04510, México

Correspondence to:

Enrique Antonio Fernandez-Torres

enrique.30065@gmail.com

**Supplementary material**

Table *S1*: Maximum average subsidence velocity comparison in previous reported areas

| States  Urban location | Max.  Vel  vert.  (cm/yr) | Comparison with previous land subsidence studies  using InSAR | | | |
| --- | --- | --- | --- | --- | --- |
|  |  | Locality^a^ | Sub.  vel.  (cm/yr) | Time  period | Reference |
| Aguascalientes/  Tepezalá^b^ | 8 | North  Aguascalientes | 8.3 | 2007-2011 | Chaussard  et al. 2014 |
| Guanajuato/  S. -L. de la Paz^b^ | 8.6 | South of San Luis  de la Paz | 8.8 | 2007-2011 | Chaussard  et al. 2014 |
| Guanajuato/  Villa de Reyes^b^ | 5.7 | West of Villa  de Reyes | 5.2 | 2007-2011 | Chaussard  et al. 2014 |
| State of Mexico/  Ciudad Nezahualcóyotl | 42.8 | Mexico City | 50 | 1995-2020 | Chaussard  et al. 2021 |
| State of Mexico/  S. -J. Guadalupe O. | 6.2 | Toluca | 7 | 2007-2011 | Chaussard  et al. 2014 |
| San Luis Potosí/  Moctezuma^b^ | 19.1 | West of Villa  Arista | 18.4 | 2007-2011 | Chaussard  et al. 2014 |
| Querétaro/  Querétaro^c^ | 1.9 | Querétaro | 1.5  5 | 2017-2020  2007-2011 | Castellazzi  et al. 2021  Chaussard  et al. 2014  2014 |
| Michocán/  S.-J. Itzícuaro | 6 | Morelia | 6.7 | 2007-2011 | Chaussard  et al. 2014 |
| Guanajuato/  Salamanca | 7.4 | Salamanca | 7.4 | 2007-2011 | Chaussard  et al. 2014 |
| Aguascalientes/  Aguascalientes | 11.1 | Aguascalientes | 15 | 2014-2016 | Cigna et al.  2021 |
| Irapuato/  SanAntonio Chico | 6.4 | Irapuato | 7.2 | 2007-2011 | Chaussard  et al. 2014 |
| Guanajuato/  San Elías | 7.3 | Celaya | 8.5 | 2007-2011 | Chaussard  et al. 2014 |
| Guanajuato/  La Aldea | 4.5 | Silao | 5 | 2007-2011 | Chaussard  et al. 2014 |
| Guanajuato/  Los Ramírez | 5 | León | 5.2 | 2007-2011 | Chaussard  et al. 2014 |
| Nayarit/  Tepic | 3.6 | Tepic | 6.8 | 2007-2011 | Chaussard  et al. 2014 |
| Michoacán/  Zamora | 9.6 | Zamora | 12.8 | 2007-2011 | Chaussard  et al. 2014 |
| Puebla/  Tlacotepec | 3.4 | Ahuacatlán | 5 | 2007-2011 | Chaussard  et al. 2014 |
| San Luis Potosí/  San Luis Potosí | 4.9 | San Luis Potosí | 3.9 | 2007-2011 | Chaussard  et al. 2014 |
| Guanajuato/  San Luis de la Paz | 2.2 | San Luis de la Paz | 4 | 2007-2011 | Chaussard  et al. 2014 |
| Jalisco/  Colonia Ninos Héroes | 5.3 | Guadalajara | 3.3 | 2007-2011 | Chaussard  et al. 2014 |
| Puebla/  Santa María  Acuexcomac | 3.4 | Puebla | 4.4 | 2007-2011 | Chaussard  et al. 2014 |
| Jalisco/  Jocotepec | 16.5 | Jocotepec | 0.89 cm/  month  ~10.7^4^ | Sep – Nov  2012 | Hernandez-  Marin et al.  2014 |
| Baja California/  Nuevo León | 9.2 | Mexicali | 12^d^ | 1994-1997 | Sarychikhina  et al. 2011 |
| Jalisco/  Ciudad Guzmán | 7.6 | Ciudad Guzmán | 2 | 2003-2010 | Brunori et al.  2015 |

^a^ As named in previous studies.

^b^ This is an agricultural area; as consequence, it was not considered in the total area, population, and households computation nor in Supplementary file1 from this work.

^c^ This locality is not in Supplementary file1 because the maximum average velocity is bellow than our previous defined threshold.

^d^ Geotechnical instrumentation.

**References**

Brunori CA, Bignami C, Zucca F, et al (2015) Ground Fracturation in Urban Area: Monitoring of Land Subsidence Controlled by Buried Faults with InSAR Techniques (Ciudad Guzmán: Mexico). In: Lollino G, Manconi A, Guzzetti F, et al. (eds) Engineering Geology for Society and Territory - Volume 5. Springer International Publishing, pp 1027–1031

Castellazzi P, Garfias J, Martel R (2021) Assessing the efficiency of mitigation measures to reduce groundwater depletion and related land subsidence in Querétaro (Central Mexico) from decadal InSAR observations. International Journal of Applied Earth Observation and Geoinformation 105:102632. https://doi.org/10.1016/j.jag.2021.102632

Chaussard E, Havazli E, Fattahi H, et al (2021) Over a Century of Sinking in Mexico City: No Hope for Significant Elevation and Storage Capacity Recovery. Journal of Geophysical Research: Solid Earth 126:1–18. https://doi.org/10.1029/2020JB020648

Chaussard E, Wdowinski S, Cabral-Cano E, Amelung F (2014) Land subsidence in central Mexico detected by ALOS InSAR time-series. Remote Sensing of Environment 140:94–106. https://doi.org/10.1016/j.rse.2013.08.038

Cigna F, Esquivel Ramírez R, Tapete D (2021) Accuracy of Sentinel-1 PSI and SBAS InSAR Displacement Velocities against GNSS and Geodetic Leveling Monitoring Data. Remote Sensing 13:4800. https://doi.org/10.3390/rs13234800

Hernandez-Marin M, Pacheco-Martinez J, Ramirez-Cortes A, et al (2014) Evaluation and analysis of surface deformation in west Chapala basin, central Mexico. Environ Earth Sci 72:1491–1501. https://doi.org/10.1007/s12665-014-3054-7

Sarychikhina O, Glowacka E, Mellors R, Vidal FS (2011) Land subsidence in the Cerro Prieto Geothermal Field, Baja California, Mexico, from 1994 to 2005: An integrated analysis of DInSAR, leveling and geological data. Journal of Volcanology and Geothermal Research 204:76–90. https://doi.org/10.1016/j.jvolgeores.2011.03.004
